# Polar Lattice‐Distorted Motifs Enable Synergy of Local Polarization/Dipole Fields for Concurrent Glyphosate Wastewater Remediation and CO Evolution

**DOI:** 10.1002/advs.202521941

**Published:** 2026-04-02

**Authors:** Daoping Chen, Huan Liu, Tengyu Liu, Jie Li, Qizhi Luo, Yan Zhang, Shengkun Li, Hu Li

**Affiliations:** ^1^ State Key Laboratory of Green Pesticides State‐Local Joint Laboratory For Comprehensive Utilization of Biomass Center For R&D of Fine Chemicals Guizhou University Guiyang Guizhou China; ^2^ Anhui Provincial Key Laboratory of Advanced Catalysis and Energy Materials School of Chemistry and Chemical Engineering State Key Laboratory of Marine Resource Utilization in South China Sea Anqing Normal University Guiyang Guizhou China; ^3^ State Key Laboratory of Marine Resource Utilization in South China Sea Department of Materials Science and Engineering Hainan University Haikou China

**Keywords:** carbon nitride, gas fuel, molecular distortion, photocatalytic degradation, wastewater remediation

## Abstract

For wastewater remediation, efficiently avoiding harmful co‐product formation while achieving carbon source recovery remains a challenge worthy of attention. Herein, we deliberately bridge a polar pyridinic moiety into carbon nitride (PDCN) to construct a lattice distortion structure with interfacial charge asymmetric polarization, achieving complete photodegradation of glyphosate (Gly) wastewater into sarcosine (>99% selectivity) and CO (1166.2 µmol g^−1^ h^−1^) within a short timeframe. The introduction of polar pyridine not only causes the C─N─C bond angle of PDCN to distort, generating a local polarization field, but also induces a dipole field, which achieves effective separation and directional transfer of photogenerated carriers, respectively. This directed electron localization enhances O_2_ adsorption by PDCN's electron‐rich pyridine regions and its conversion into reactive •OH for cleaving C─P bonds. Furthermore, PDCN's electron‐deficient heptazine region activates Gly's α‐C─H bond via electrostatic interactions with phosphate groups, promoting the selective cleavage of C─P bonds in Gly and avoiding the formation of toxic aminomethylphosphonic acid. Glycine and other co‐products show no significant impact on the growth of organisms like fathead minnows and mung beans. This work establishes a viable perspective for efficient wastewater purification and carbon recovery enabled by the synergistic interaction of localized polarization and dipole fields.

## Graphical abstract

Photocatalytic treatment of glyphosate herbicide in agricultural wastewater is achieved through the cooperative effect of the local polarization field and dipole field mediated by lattice‐distorted carbon nitride. Glyphosate is completely degraded via selective C─P bond cleavage with a CO evolution rate of 1166 µmol g^−1^ h^−1^. This protocol offers a viable approach for efficient wastewater purification and carbon upcycling.

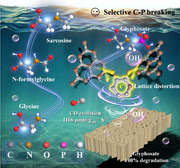

## 1 Introduction

Organophosphorus pesticides represent a class of highly effective and broad‐spectrum agrochemicals extensively utilized worldwide, playing a pivotal role in agricultural pest management [1]. As a predominant non‐selective herbicide, glyphosate (Gly) is projected to reach an annual global consumption of 740 000 to 920 000 metric tons by 2025 [2, 3]. Excessive application and improper management of Gly not only jeopardize flora and fauna but also contaminate aquatic systems, terrestrial environments, and atmospheric compartments through multiple pathways [4–6]. Currently, the widely adopted methods for wastewater remediation include biodegradation [7, 8], adsorption [9, 10], and chemical oxidation [11, 12]. However, these treatment technologies present various limitations, in which biodegradation exhibits slow reaction kinetics, the selection and regeneration of adsorbent materials constitute key technical challenges, and chemical oxidation may induce secondary pollution. Moreover, such systems typically degrade Gly into greenhouse gas CO_2_ in a non‐selective manner, which is detrimental to sustainable development. The degradation pathways of Gly are generally divided into two: (i) Initial C─P bond scission of Gly yields sarcosine (Sar) or glycine, with CO_2_ and orthophosphate as end products; (ii) The alternative is to initially sever the Gly‐end C─N bond to form aminomethylphosphonic acid (AMPA) [13]. It is noteworthy that AMPA exhibits toxicity comparable to its parent compound Gly, with prolonged soil half‐life and pronounced bioaccumulation potential [14]. Consequently, the development of eco‐friendly water purification strategies featuring selective degradation and carbon resource utilization remains a predominant research focus in current scientific investigations.

Advanced oxidation processes (AOPs) are capable of achieving in situ degradation of organic pollutants through transient reactive species generated during reactions [15]. Among them, photocatalytic technology has drawn wide attention due to its mild reaction conditions, strong oxidative ability, and being free of secondary pollution [16]. Conventional semiconductor photocatalysts (e.g., BiVO_4_, NiSnO_3_, Bi‐MOF, and Ag‐TiO_2_) [17–20] can be modified to effectively degrade Gly by metal doping, vacancy engineering, covalent molecular integration for metal organic frameworks (MOFs) construction, or heterojunction formation. However, the lack of specific active sites and reactive species often leads to non‐selective degradation of Gly, accompanied by various negative issues such as CO_2_ emission and inevitable metal leaching. In this context, non‐metallic semiconductor carbon nitride (CN) organic materials have drawn substantial attention because of their adjustable bandgap, excellent chemical and thermal stability, and facile synthesis [21, 22]. Typically, the highly symmetric heptazine ring structure in CN causes the photogenerated charge carriers to recombine rapidly, which significantly hinders its photocatalytic efficiency [23]. Current modification strategies for CN primarily encompass (i) atomic doping, (ii) molecular engineering, (iii) defect construction, and (iv) heterostructure fabrication [24–27], which have been demonstrated to significantly enhance CN's light‐harvesting efficiency, charge carrier separation capability, and surface reaction kinetics. Recent studies have revealed that distorted 2D graphene monolayer structures exhibit greater stability compared to boundary‐free planar configurations, with spontaneous distortion formation occurring in CN frameworks to mitigate repulsive interactions between nitrogen lone electron pairs [28, 29]. Partial organic molecular modifications induce spontaneous distortion of the CN 2D structure into a 3D configuration, facilitating n‐*π* ^*^ electron hopping. This asymmetric polarization structure induces a localized polarizing field that accelerates intermolecular charge transfer, reduces the bandgap, and enhances light absorption capacity [23]. Inspired by these findings, tailored modification of CN semiconductors with strongly electronegative organic units can result in the generation of a dipole field, which is anticipated to further promote n‐*π* ^*^ electronic transition, thereby accelerating the formation of asymmetric polarization structures that are more conducive to redox reactions.

In this work, we construct a pyridine‐modified carbon nitride (PDCN) catalyst to exhibit a spontaneous C─N─C bond angle distortion (20.78°), disrupting the original coplanarity of CN. The synergistic effect of localized polarization and dipole fields accelerates interfacial charge transfer, which not only promotes spatial dissociation of photogenerated excitons but also induces their directional localization, thereby rapidly establishing electrostatic interaction with Gly. Charge carrier localization further effectively enhances the electrostatic interaction, which in turn accelerates C─H bond scission during the proton‐coupled electron transfer (PCET) process, thus driving selective C─P bond scission for environmentally benign upgrading of carbon source to CO. Moreover, the superior conduction band potential of the PDCN photocatalyst facilitates the in situ production of critical reactive oxygen species (ROS) •OH. Structural engineering is shown to enhance the dipole moment of CN from 0.04 D to 2.84 D for PDCN, as revealed by dipole moment computation and Kelvin probe force microscopy (KPFM), demonstrating the synergistic role of the dipole field and localized polarized electric field in accelerating charge separation. Environmental sustainability was evaluated using the toxicity estimation software tool (T.E.S.T) and validated by mung bean toxicity tests. It is suggested that photocatalytic purification of Gly wastewater could circumvent toxic byproducts during the upcycling process. This study offers new perspectives for concurrent high‐performance wastewater remediation and in situ carbon utilization of organic pollutants.

## 2 Results and Discussion

### 2.1 The Photocatalyst Preparation and Characterization

**FIGURE 1:**
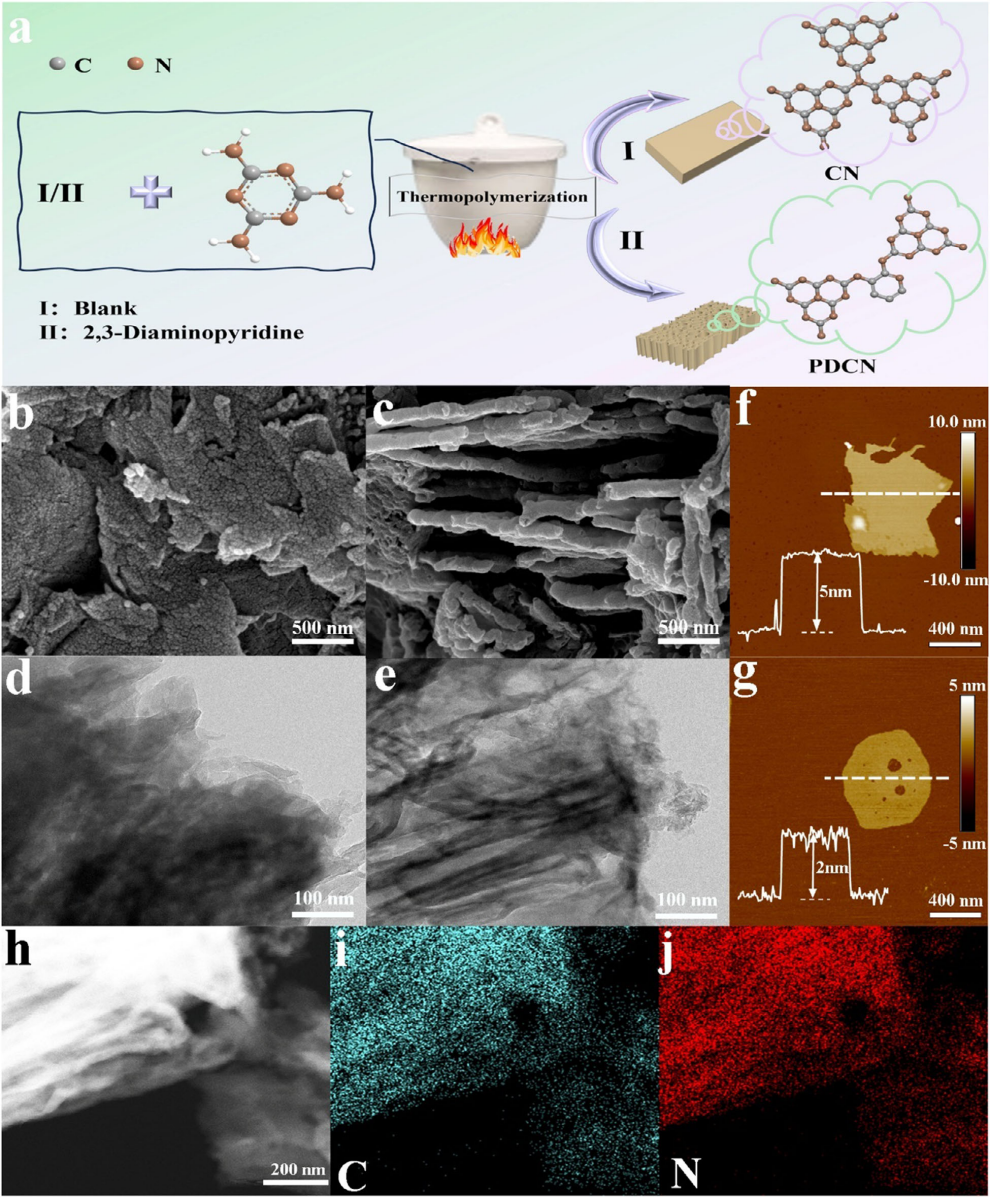
(a) Schematic of the thermal condensation polymerization process for preparing CN and PDCN. SEM images of (b) CN and (c) PDCN. TEM images of (d) CN and (e) PDCN. AFM images of (f) CN and (g) PDCN. (h‐j) HAADF STEM and EDS mapping images of PDCN.

CN and PDCN catalysts were prepared by a straightforward molecular self‐assembly thermal polycondensation method (Figure 1a). Scanning electron microscopy (SEM) and transmission electron microscopy (TEM) provide insights into the catalysts’ morphology and microstructure. CN exhibits a layered bulk structure (Figure 1b) after direct melamine calcination, whereas PDCN's coral‐like tubular structure (Figure 1c) results from pyridine group‐induced steric hindrance that promotes tubular architectures [30]. TEM observations reveal that CN exfoliates into multilayered bulk sheets (Figure 1d), while PDCN exhibits a hollow microtubular morphology (Figure 1e), which is consistent with the SEM results. Compared to pristine CN (Figure 1f), the PDCN sample exhibits more pronounced curled edges (Figure 1g), which results from its ultrathin thickness as evidenced by atomic force microscopy (AFM) images. In comparison to pristine CN (∼5 nm), PDCN displays a smaller average thickness of around 2.0 nm. Moreover, high‐angle annular dark field scanning transmission electron microscopy (HAADF STEM) image and energy‐dispersive spectroscopy (EDS) mappings of PDCN (Figure 1h–j) clearly show that C and N elements are evenly distributed in the catalyst. The C/N molar ratio was determined by X‐ray photoelectron spectroscopy (XPS). As shown in Table S1, PDCN exhibits a C/N molar ratio value of 0.90, higher than that of CN (0.86), mainly due to the incorporation of pyridine units.

N_2_ adsorption‐desorption isotherms provided insights into the catalysts’ surface area and pore structure (Figure S1a). Both CN and PDCN catalysts exhibit type IV isotherms, with specific surface areas (S_BET_) of 12.7 and 48.5 m^2^ g^−1^, respectively. Notably, the surface area of PDCN is approximately four times greater than that of CN. This result is consistent with the morphological observations from SEM (Figure 1b,c), further confirming that the coral‐like tubular architecture of PDCN effectively enhances its surface area. The inset in Figure S1 presents the corresponding pore size distribution profiles, indicating average pore diameters of 13.7 and 26.3 nm for CN and PDCN, respectively, which confirms the mesoporous structure of both catalysts. The larger specific surface area and more abundant pore structure expose ample active sites, thus driving marked improvements in photocatalytic performance.

**FIGURE 2:**
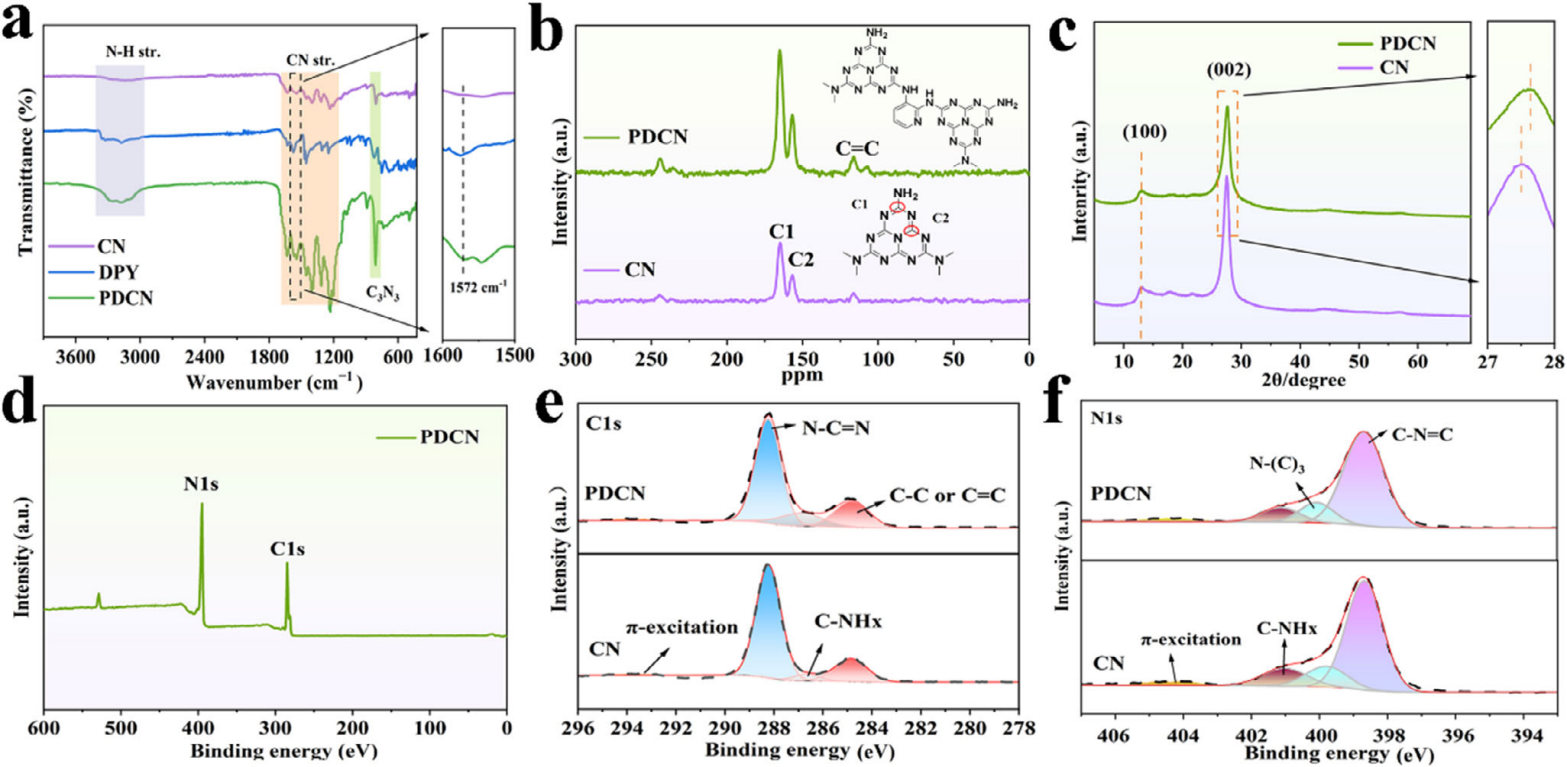
(a) FTIR spectra, (b) Solid‐state ^13^C NMR spectra, and (c) XRD patterns of CN and PDCN photocatalysts. (d) XPS survey spectrum of PDCN. High‐resolution XPS spectra of (e) C 1s and (f) N 1s for CN and PDCN photocatalysts.

Fourier‐transform infrared (FTIR) measurements were additionally employed to assess the molecular structural properties of the as‐synthesized catalyst (Figure 2a). When analyzing the FTIR spectra of CN and PDCN, three distinct absorption bands appear for CN at 806 cm^−1^ (from the inherent breathing mode of heptazine ring units), 1160‒1680 cm^−1^ (stretching and bending motions within C─N heterocycles), and 2960‒3400 cm^−1^ (the N─H stretching movements on the surface) [19]. The incorporation of pyridine units in PDCN results in the preservation of all characteristic vibrational peaks observed in CN, indicating that the covalent integration of 2,3‐diaminopyridine (DPY) maintains the structural framework of carbon nitride without significant alteration. Furthermore, the intensified N─H characteristic peak of the PDCN catalyst results from the increased population of N─H species caused by the covalent incorporation of pyridine units into the framework through C─N─C bonds upon introduction of DPY. The enhancement of PDCN's characteristic peaks is primarily due to the overlapping of most vibrational peaks of DPY with those of CN. Notably, the PDCN catalyst exhibits a new peak at 1572 cm^−1^, characteristic of the aromatic C═C stretching in the bridged pyridine unit [31]. This finding confirms that pyridine was effectively introduced into the CN framework.

The detailed structural features of the catalysts were further investigated using the solid‐state ^13^C nuclear magnetic resonance (^13^C NMR) spectroscopy. Two distinct peaks are observed for CN at 164.5 and 156.6 ppm (Figure 2b). The peak at 164.5 ppm originates from sp^2^‐hybridized carbon atoms (C1) connected to terminal ─NH_2_ groups, while the peak at 156.6 ppm is associated with sp^2^‐hybridized carbon atoms (C1) attached to bridging nitrogen atoms (C─N_3_) within heptazine units [32]. The signal peak intensities of C1 and C2 in PDCN are comparatively higher than those in CN, which is attributed to the formation of novel C─N─C chemical bonds between the pyridine moiety and the CN framework [30]. This results in substantial alterations in the local carbon environment of CN. In contrast to CN, the PDCN catalyst displays two intense peaks at 116.1 and 107.2 ppm, ascribed to aromatic C═C bonds, indicating that pyridine ring units are embedded within the heptazine‐based framework [33]. Furthermore, the solid‐state ^1^H nuclear magnetic resonance (^1^H NMR) spectra (Figure S1b) reveal that the pyridine ring protons exhibit characteristic paramagnetic shift resonance [34], presenting three pairs of signals: α_a_/α_b_ (65.6, −49.2 ppm), β_a_/β_b_ (38.4, −20.7 ppm), and γ_a_/γ_b_ (30.1, −14.0 ppm). The signal at 8.0 ppm is assigned to the ─NH/─NH_2_ groups, which aligns with the FTIR spectral results (Figure 2a), further confirming the successful synthesis of PDCN.

The presence of a pair of diffraction signals at 12.87° and 27.51° is a consistent feature in the X‐ray diffraction (XRD) profiles of two catalysts (Figure 2c). These peaks originate from the (100) and (002) lattice planes of CN, with the former corresponding to in‐plane compression of heptazine units and the latter to interlayer stacking [19]. The similarity between the diffraction pattern of PDCN and that of CN indicates that the primary structural framework of CN remains unaltered despite the bridging of pyridine rings, which is in good agreement with the results of FTIR characterization (Figure 2a). Following pyridine modification, the (002) diffraction peak exhibited significant attenuation, indicating a reduction in the crystallinity of the CN framework [32]. Furthermore, a close‐up view of the XRD pattern for the PDCN catalyst shows that the (002) diffraction peak shifts to a higher angle relative to pristine CN, an observation attributed to the reduced interlayer spacing upon pyridine incorporation [24]. Note that a stronger interlayer cohesion can boost charge transport and cut the interfacial energy barrier in the layered material.

The XPS survey spectra of PDCN and CN show two intense features at 397.6 and 287.5 eV (Figure 2d; Figure S2), attributed to N 1s and C 1s, respectively, which is consistent with the EDS mapping results (Figure 1h–j). Both PDCN and CN catalysts exhibit four comparable characteristic peaks in their C 1s XPS spectra (Figure 2e) at 284.80 (C─C/C═C), 286.87 (C─NH_x_), 288.24 eV (N─C═N), and 293.46 eV (π excitation) [25]. The N 1s XPS analysis shows (Figure 2f) characteristic peaks of PDCN and CN at 398.67 (C─N═C), 399.19 (N─(C)_3_), 401.01 (C─NH_x_), and 404.51 eV (corresponding to π‐excitation) [26]. Notably, the C─NH*_x_* peak intensity is stronger in the C 1s spectrum of PDCN than in that of CN. It is suggested that thermal polymerization between the pyridine moiety and heptazine of PDCN increases the number of N─H bonds, which is in line with FTIR analysis results (Figure 2a). Notably, the N─(C)_3_ peak in the N 1s spectrum of PDCN is weaker than that of CN, which is caused by the introduction of pyridine with high proton density [35]. Additionally, the N─(C)_3_ peak (399.19 eV) exhibits a distinct shift toward a higher binding energy (400.07 eV). This shift points to diminished electron density surrounding nitrogen atoms, a phenomenon that may arise from the induced dipole field generated by polar pyridine units integrated into the PDCN framework [36]. These experimental results strongly demonstrate the successful synthesis of PDCN.

Density functional theory (DFT) modeling was employed to carry out geometric optimization of CN and PDCN fragments (Figure S3), and the results show that the heptazine rings in CN have C─N─C dihedral angles of 0°, 0.036°, and 0.037°, which indicate a state of nearly perfect coplanarity. In contrast, the C─N─C dihedral angles between the heptazine and pyridine rings in PDCN are 1.11° and 20.78°, respectively, demonstrating significant intramolecular torsion. Pyridine embedding causes structural distortion, which disrupts the symmetric arrangement of CN's local regions.

### 2.2 Photoelectric Property

**FIGURE 3:**
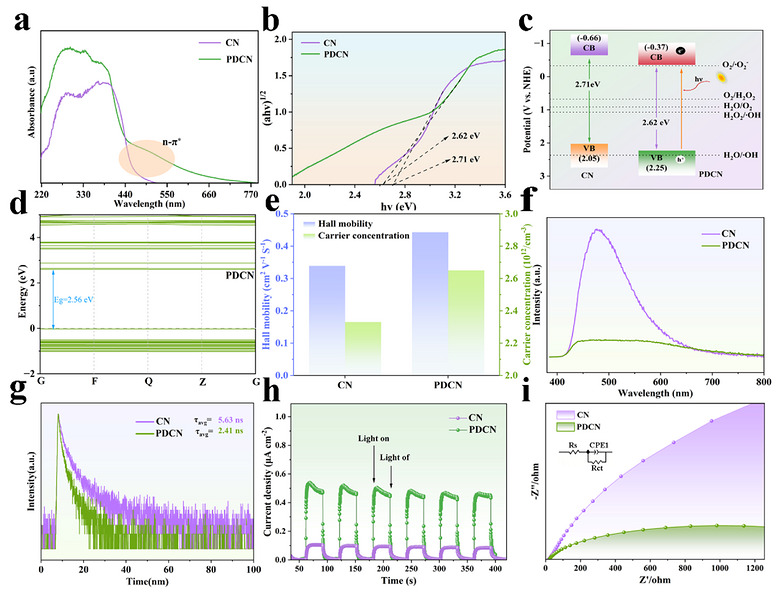
(a) UV–Vis DRS spectra, (b) Tauc plots, (c) band structure diagrams, (d) DFT‐calculated band diagrams, (e) hall effect parameters, (f) PL spectra, (g) TRPL spectra, (h) photocurrent potential curves measured under visible light irradiation, and (i) EIS Nyquist plots of CN and PDCN photocatalysts.

Through molecular engineering, the photocatalyst's electronic configurations and light absorption characteristics can be precisely tuned. As shown in Figure 3a, ultraviolet‐visible diffuse reflectance spectroscopy (UV–Vis DRS) indicates that PDCN presents a CN‐specific absorption feature spanning the 220‒460 nm region, originating from the *π*–*π* ^*^ transition in conjugated heterocyclic frameworks of CN [37]. PDCN demonstrates a color transition from yellow to brown (Figure S4), with a pronounced red shift of the characteristic absorption peaks, which is attributed to the π‐conjugation network extended by the aromatic pyridine moiety [38]. Compared with the CN catalyst, PDCN exhibits a distinct broad peak at 480 nm, corresponding to an intrinsic n‐*π* ^*^ electron transition. Due to the fully symmetric planar units, n‐*π* ^*^ transitions are typically forbidden in CN, suggesting that the introduction of pyridine rings leads to the formation of an asymmetric planar structure within CN, and that the n‐*π* ^*^ electronic transition causes an uneven local charge distribution, resulting in a local polarization field [32]. This local polarization field provides a driving force for the dissociation of photogenerated excitons, facilitating the separation of electron‐hole pairs at the molecular level [39]. Band gap values (Eg) can be estimated from Tauc plots, which were obtained using the Kubelka–Munk function (Figure 3b). Compared to CN (2.71 eV), PDCN exhibits a reduced band gap (2.62 eV), making it more conducive to electron transitions. VB‐XPS analysis was conducted (Figure S5), with the maximum valence band (E_VBM_) determined as 2.49 eV for PDCN versus 2.29 eV for CN. Valence band potentials (E_VB_) for PDCN and CN, calculated via E_VB_ = E_VBM_ + Φ − 4.44 eV (Equation S1), are estimated to be 2.25 and 2.05 eV, respectively. A higher E_VB_ value points to greater oxidative capacity of the holes in the PDCN photocatalyst [40]. On this basis, the conduction band (CB) positions of PDCN and CN catalysts were determined according to E_g_ = E_CB_ + E_VB_ (Equation S2; Figure 3c). The bridging of pyridine rings directly brings about a positive shift of the CB and a reduction in the band gap. The concurrent movement of the CB and VB in the same direction is a result of the quantum confinement effect, which implies that the pyridine ring incorporation suppresses the recombination process between photogenerated electrons and holes (e^−^–h^+^), thus enhancing the charge separation efficiency [41]. The Eg values from DFT band structure simulations (Figure 3d; Figure S6) are 2.70 eV for CN and 2.65 eV for PDCN, revealing the band gap reduction in PDCN upon introduction of pyridine, in good agreement with characterization results. Hall effect measurements were performed to quantify the carrier mobility and concentration (Figure 3e). PDCN shows a higher Hall mobility (0.44 cm^2^ V^−1^ s^−1^) and carrier concentration (2.65 × 10^12^ cm^−3^) compared to CN (0.34 cm^2^ V^−1^ s^−1^, 2.33 × 10^12^ cm^−3^, respectively), indicating that pyridine modification of CN effectively improves charge generation and transport. Mott–Schottky plots (M‐S, Figure S7) confirm the n‐type nature of both samples (Figure 3c), with flat band potentials of −0.66 V (vs. NHE) for CN and −0.37 V (vs. NHE) for PDCN, matching the conduction band positions. These findings demonstrate that the enhanced carrier dynamics and suitable reduction potential contribute to the superior photocatalytic activity of PDCN.

For the purpose of evaluating the separation performance of charge carriers in PDCN and CN catalysts, photoluminescence (PL) and time‐resolved photoluminescence (TRPL) spectra were collected. CN exhibits a PL emission peak at 476 nm (Figure 3f), which arises from radiative recombination as photoexcited e^−^–h^+^ pairs transition from a higher‐energy excited state back to the lower‐energy ground state. PDCN exhibits a reduced PL signal compared to CN, indicating that the radiative recombination of photoexcited carriers has been inhibited. This can be ascribed to the distorted C─N─C bond angle that mediates a localized polarization field, facilitating n‐π^*^ electronic transition and promoting the separation of photoexcited free carriers. Furthermore, the average fluorescence lifetime (τ_avg_) was obtained by fitting the TRPL decay curves (Figure 3g; Table S2). PDCN has a shorter τ_avg_ (2.41 ns) in comparison to CN (5.63 ns), suggesting higher charge carrier mobility in the former. This enhancement is attributed to the dipole field induced by the polar pyridine unit modification, which increases the mobility of photogenerated carriers. Overall, the PL and TRPL results demonstrate that covalent incorporation of the pyridine moiety facilitates charge transfer while inhibiting e^−^–h^+^ recombination.

Transient photocurrent response and electrochemical impedance spectroscopy (EIS) were further employed to assess the photoelectrochemical properties of the CN and PDCN. The photocurrent‐potential curves in Figure 3h show that PDCN possesses a higher photocurrent density than CN. The EIS Nyquist plot (Figure 3e) demonstrates that a more compact arc radius is observed for PDCN, a characteristic that points to a lower magnitude of charge transfer resistance. These results collectively indicate that PDCN possesses faster charge carrier mobility and higher separation efficiency, compared to CN [26].

**FIGURE 4:**
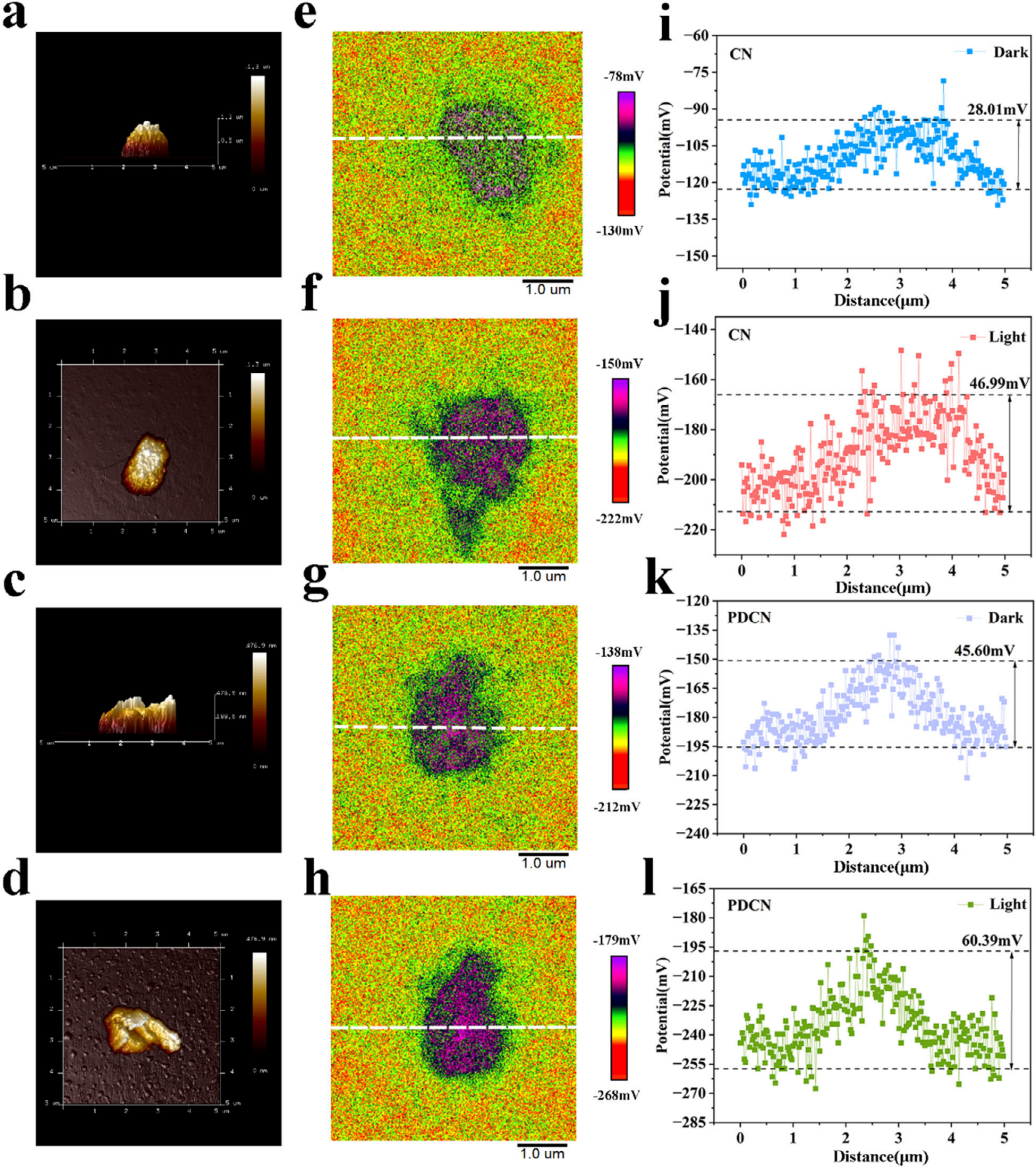
AFM 3D morphology images of (a,b) CN and (c,d) PDCN. In situ KPFM surface potential images of (e,f) CN and (g,h) PDCN under dark (Dark) and light (Light) conditions, respectively. Contact potential difference of (i,j) CN and (k,l) PDCN obtained by averaging along the vertical direction at the in situ KPFM cursor position.

With the aim of further examining the spatial charge separation capability of CN and PDCN catalysts under light irradiation, in situ Kelvin probe force microscopy (KPFM) was utilized to determine the contact potential difference (CPD) under both illuminated and dark conditions (Figure 4). By measuring along the vertical direction within the regions delineated by the in situ KPFM cursor lines (Figure 4e–h), the CPD values of PDCN and CN under dark and light conditions were obtained (Figure 4i–l). Under dark conditions, the CPD of PDCN (45.60 mV) is greater than that of CN (28.01 mV). This difference suggests that PDCN possesses a more pronounced surface potential compared to CN. Notably, both catalysts exhibit a further light‐induced increase in CPD from dark to illuminated conditions, with the most pronounced change observed on the PDCN surface under illumination (60.39 mV, Figure 4i–l). This significant light‐induced CPD variation demonstrates that PDCN exhibits superior charge separation efficiency, leading to a higher concentration of photogenerated charges accumulating at the surface, which is consistent with the characterization results of PL and TRPL (Figure 3f,g) [42]. The results indicate that the dipole field induced by the polar pyridine moiety works synergistically with the local polarized electric field of the asymmetric structure to amplify spontaneous polarization, thereby improving the dissociation performance of photoexcited e^−^–h^+^ pairs.

### 2.3 Photocatalytic Activity

**FIGURE 5:**
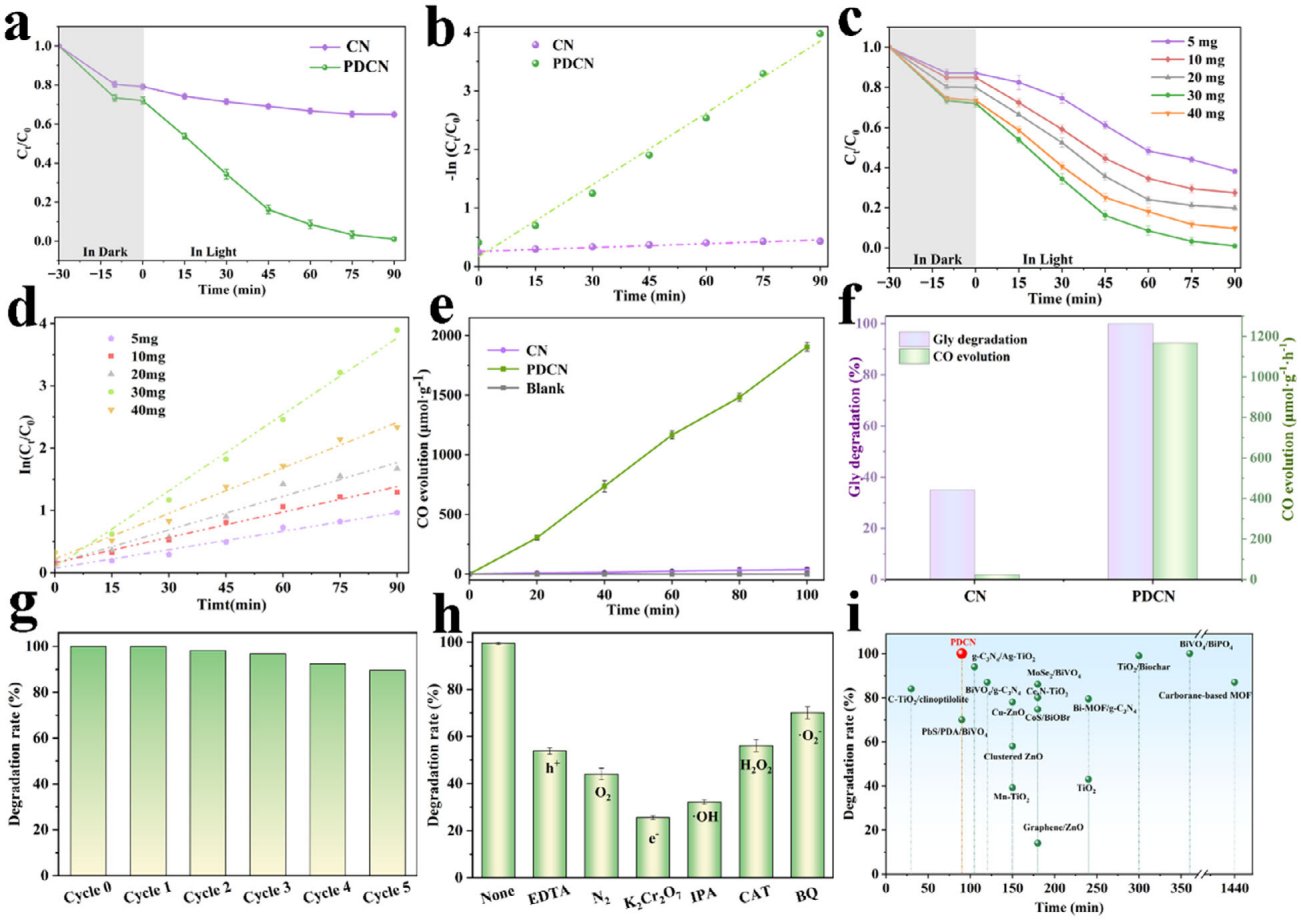
(a) Gly degradation profiles over CN and PDCN catalysts and (b) associated kinetic curves. (c) Gly removal efficiency versus PDCN catalyst dosage and (d) corresponding kinetic curves. (e) Time‐dependent CO production rates of CN and PDCN catalysts. (f) Correlation between Gly degradation rate and CO evolution rate over PDCN catalyst. (g) Recycling tests of the PDCN catalyst. (h) Active species quenching experiments. (i) Performance comparison between the current work and previous studies. (Reaction Conditions: 30 mg catalyst, 7.1 mmol/L initial Gly concentration, irradiation with 300 W visible‐light xenon lamp, and pH = 7, with triplicate experiments).

Given the excellent photoelectric performance of the developed catalyst, the photodegradation behavior of Gly was further investigated under simulated sunlight (Figure 5). Initially, the influence of different catalysts on the Gly degradation reaction activity was systematically evaluated. Within 1.5 h, the degradation rates for PDCN and CN reach 100% and 35.06%, respectively (Figure 5a). Furthermore, it is found that the PDCN catalyst exhibits a kinetic rate constant (k_obs,_ k_obs_ = 0.0409 min^−1^), exceeding that of the CN catalyst (k_obs_ = 0.0022 min^−1^) by about 18.6 times (Figure 5b). Notably, even after normalization by the specific surface (k_norm_, k_norm_ = k_obs_/S_BET_), PDCN (8.4 × 10^−4^ g min^−1^ m^−2^) still displays a rate constant 4.94 times higher than that of CN (1.7 × 10^−4^ g min^−1^ m^−2^). This clearly demonstrates that enhanced photocatalytic activity originates from the intrinsic electronic structure, rather than merely from the surface area effect. The near‐perfect symmetrical structure of CN results in a high carrier recombination rate upon photoexcitation, which impedes ROS generation and directly leads to its poor Gly degradation capability. With increasing dosage of the PDCN catalyst (Figure 5c), the photodegradation of Gly exhibits varying degrees of enhancement within 1.5 h. However, further enhancement is suppressed beyond 30 mg, demonstrating optimal complete degradation efficacy. Excessive addition may cause the blockage of active sites, thereby reducing degradation activity. Further experiments illustrate that they all follow first‐order kinetics (Figure 5d), with 30 mg of the catalyst demonstrating the optimal kinetic rate constant (0.0409 min^−^^1^).

To assess how initial pH influences Gly removal efficiency, the solution pH was adjusted using dilute HCl and NaOH solutions, and the corresponding impact was investigated (Figure S8). PDCN displays relatively stable photodegradation activity when tested over the pH range of 3 to 9, a property that confers an advantage for its use in real wastewater systems (where pH is generally 4‒5) for the practical degradation of Gly [43]. The observed reduction in degradation efficiency may result from the fact that electrostatic repulsion occurs between Gly molecules and the catalyst surface under alkaline conditions. Specifically, the catalyst surface carries a negative charge in alkaline environments, while Gly molecules are negatively charged due to their phosphate functional groups [44]. This mutual electrostatic repulsion hinders the degradation process. CO is widely utilized as a combustible gaseous fuel in the energy sector [45]. In this study, CO evolution during Gly degradation was investigated using the synthesized catalysts. Gas chromatography analysis (Figure 5e,f) reveals that under complete Gly degradation, the CO production rate of PDCN reaches 1166.2 µmol g^−1^ h^−1^, which is approximately 50 times higher than that of CN (23.26 µmol g^−1^ h^−1^). The CO yield of CN is almost negligible, consistent with its poor photosensitivity and limited carrier separation efficiency.

To evaluate the operational stability of the PDCN catalyst, cycling tests were conducted, demonstrating a degradation rate of 85.63% after five consecutive cycles while still maintaining a high degradation efficiency (Figure 5g). The slight decrease in activity may be attributed to the accumulation of intermediates from Gly degradation at the active sites, which hinders the access of the reactant Gly [46]. Furthermore, the crystalline structure and surface chemical states of PDCN before and after the reaction were probed by XRD and XPS (Figures S9 and S10). The results indicate no significant structural alterations between the fresh and recycled catalysts. These experimental and characterization findings collectively affirm the excellent recyclability and stability of PDCN. For the identification of active species influencing Gly degradation by PDCN, scavenging experiments were undertaken. As revealed in Figure 5h, when isopropanol (IPA) is added as a scavenger, the reaction is markedly suppressed. It is indicated that Gly degradation is driven primarily by •OH as an active species, which also plays a critical role in CO generation. Furthermore, the significant inhibition of Gly degradation under N_2_ atmosphere indicates that O_2_ is a critical precursor for ROS generation, while the residual 44% removal efficiency suggests that a portion of reactive oxygen species may originate from H_2_O oxidation. Additionally, when catalase (CAT) and benzoquinone (BQ) were introduced as trapping agents targeting H_2_O_2_ and •O_2_^−^, respectively, the observed non‐negligible suppression of Gly degradation implies that both H_2_O_2_ and •O_2_^−^ play indispensable roles during the in situ formation of •OH reactive oxygen species.

Previous literature on various catalytic systems for Gly degradation [14, 18–20, 44, 47–57] has primarily employed metal‐based photocatalysts such as g‐C_3_N_4_/Ag‐TiO_2_, Mn‐TiO_2_, and Cu‐ZnO. Non‐metallic materials like MOFs have also been developed, achieving 80%‒90% Gly removal under visible light irradiation (Figure 5i). However, these systems often require prolonged reaction times (12‒60 h) and typically result in non‐selective degradation of Gly into a mixture of toxic small molecules and greenhouse gases such as CO_2_, along with intermediates like AMPA, while the recycling of carbon resources is frequently overlooked. In contrast, the metal‐free carbon‐nitrogen‐based PDCN catalyst developed in this study utilizes asymmetric surface electron dynamics to accomplish complete Gly degradation within a shorter timeframe (1.5 h), bypassing AMPA formation. Furthermore, the CO evolution rate exceeds that of recent advanced CO_2_ reduction systems (Figure S11), demonstrating exceptional redox performance. These results confirm the mechanistic feasibility of the PDCN catalytic system for rapid and environmentally benign Gly removal, as well as for directed photoconversion into gaseous fuels.

### 2.4 Theoretical Calculation and Reaction Mechanism

**FIGURE 6:**
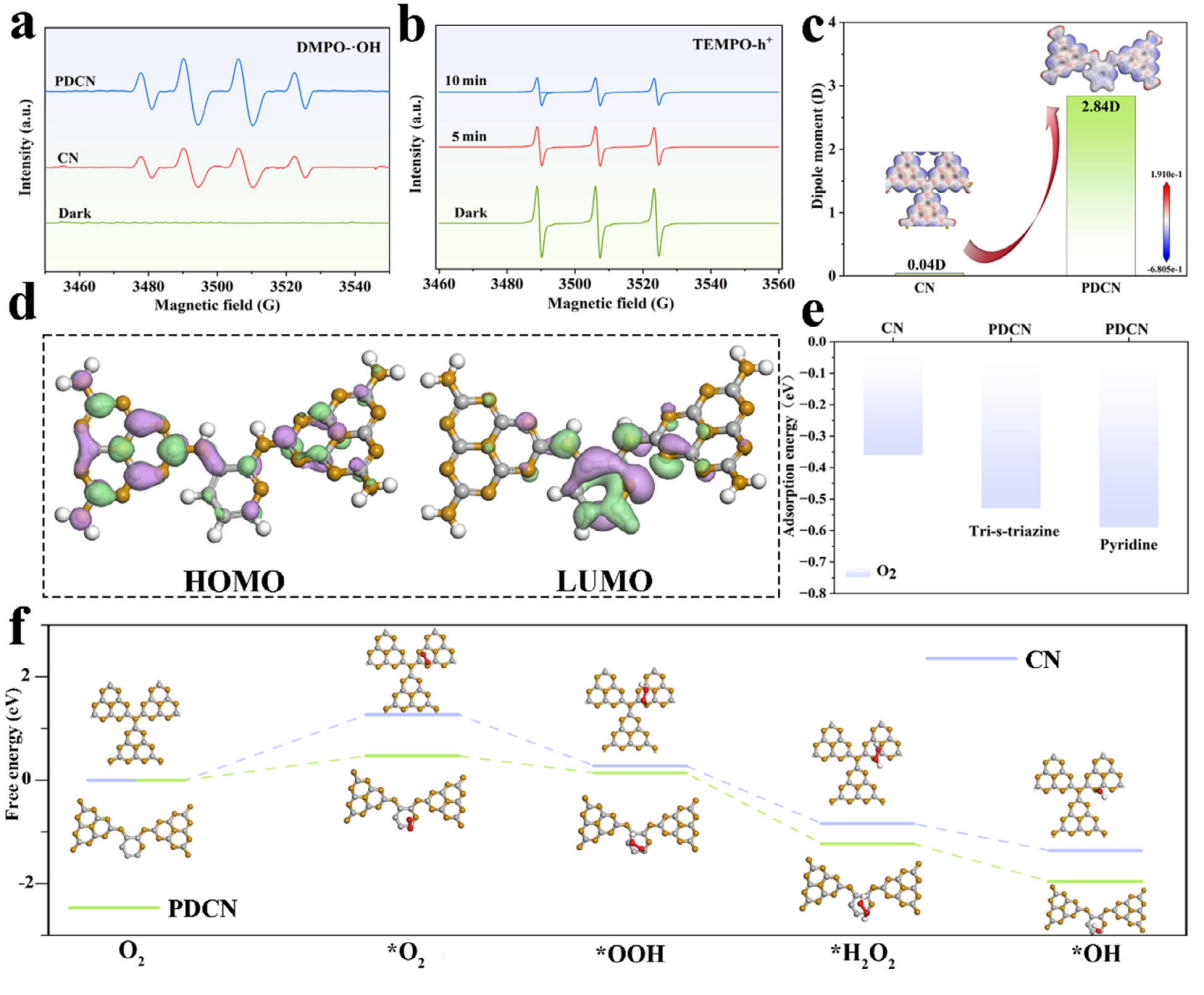
(a) DMPO‐•OH ESR spectra of CN and PDCN. (b) DMPO‐h^+^ ESR spectra of PDCN. (c) Electrostatic potential surfaces and dipole moments of CN and PDCN. (d) HOMO and LUMO electron density distributions of PDCN. (e) Adsorption energies for Gly on CN and PDCN. (f) Free energy diagram of the oxygen reduction reaction on CN and PDCN.

The reactive species in the system were further investigated using in situ electron spin resonance (in situ ESR) tests (Figure 6a,b; Figure S12). 5,5‐Dimethyl‐1‐pyrroline N‐oxide (DMPO) forms DMPO‐•OH and DMPO‐•O_2_^−^ adducts with •OH and •O_2_^−^, respectively. The findings indicate that both PDCN and CN catalysts produce •OH, with the PDCN catalyst exhibiting a more pronounced characteristic 1:2:2:1 quartet signal (Figure 6a). Furthermore, the •O_2_^−^ signal intensity in the PDCN catalyst increases with prolonging the irradiation time (Figure S12). With 2,2,6,6‐tetramethylpiperidine‐1‐oxyl (TEMPO) serving as a spin trapping agent for h^+^ (Figure 6b), the ESR signal is observed to gradually diminish over time under illumination, indicating increased generation of h^+^. Since e^−^ and h^+^ are generated in pairs, the ESR signal trend of e^−^ resembles that of h^+^. These findings demonstrate that pyridine modification enhances the photoresponsiveness and local charge density of PDCN, thereby facilitating the directional generation of ROS (•OH). This is consistent with the results of the quenching experiment (Figure 5h).

The above in situ ESR characterization and quenching experiments indicate that O_2_ is crucial for the formation of ROS •OH. Therefore, the O_2_ adsorption behavior of PDCN and CN was investigated using O_2_ temperature‐programmed desorption (O_2_‐TPD), and the findings are shown in Figure S13. The PDCN catalyst exhibits a stronger O_2_ desorption signal at 116 °C compared to CN (106 °C), indicating its superior O_2_ adsorption capacity from the solution and lower activation energy barrier for O_2_ activation, which facilitates the formation of •OH [58]. According to the band structure positioning and potential diagram of the catalysts (Figure 3c), the PDCN catalyst is capable of oxidizing H_2_O to generate O_2_ in the reaction system (Figure S13). Therefore, the O_2_ content was monitored during the reaction. After 45 and 90 min of reaction, the detected O_2_ content (corresponding to peak areas of 1 167 236 and 1 179 612, respectively) remains almost unchanged (Figure S14). This result suggests that O_2_ can be continuously generated in the system to provide a steady supply for catalytic conversion, thereby facilitating the production of ROS (•OH), which aligns with the quenching experiment results (Figure 5h). Specifically, even after the dissolved oxygen in the reaction solution is removed by N_2_, the degradation rate of Gly still reaches 44%, indicating that part of the O_2_ originates from the oxidation of H_2_O.

To explore the influence of the bridging of the pyridine skeleton on the carrier distribution of CN, frontier molecular orbital DFT calculations on CN and PDCN were conducted. The surface electrostatic potentials of CN and PDCN were also computed. The results indicate that the sp^2^ N (C─N═C) and pyridine nitrogen sites in both CN and PDCN exhibit relatively low electrostatic potentials (Figure 6c), rendering them more susceptible to nucleophilic attraction. Upon introduction of the pyridine moiety, the calculated dipole moment increases from 0.04 D for CN to 2.84 D for PDCN (Figure 6c), which can drive photogenerated electrons and holes to migrate directionally in opposite directions, thus promoting charge separation and transport [59]. Therefore, DFT‐based frontier molecular orbital calculations of PDCN reveal (Figure 6d) that its highest occupied molecular orbital (HOMO) is predominantly localized in the heptazine ring region, whereas the pyridine ring region acts as the main distribution area for the lowest unoccupied molecular orbital (LUMO), which stands in sharp contrast to CN (Figure S15). The HOMO and LUMO are distinctly separated for PDCN due to the synergy between the local polarization field and dipole‐induced charge polarization, which promotes electron‐hole separation and directional transfer, respectively. Further investigation into the adsorption performance and sites of O_2_ on PDCN and CN catalysts was conducted by DFT calculations. The adsorption models of O_2_ are illustrated in Figure 6e and Figure S16. The O_2_ adsorption energies on CN, the pyridine ring of PDCN, and the heptazine ring of PDCN were calculated to be −0.35, −0.59, and −0.53 eV, respectively (Figure 6e). The data show that O_2_ preferentially adsorbs on the pyridine ring of PDCN, suggesting that pyridinic nitrogen sites facilitate O_2_ adsorption/activation for •OH generation, in line with the surface electrostatic potentials results (Figure 6c). The Gibbs free energy for •OH generation from O_2_ on CN and PDCN was also calculated (Figure 6f). The rate‐determining step is identified as O_2_ adsorption, where PDCN exhibits a lower energy barrier (0.47 eV) compared to CN (1.27 eV). It is demonstrated that the introduction of the pyridine moiety further promotes O_2_ adsorption/activation, which promotes the proton‐coupled electron transfer process for •OH generation [60], aligning with the O_2_‐TPD data (Figure S13).

**FIGURE 7:**
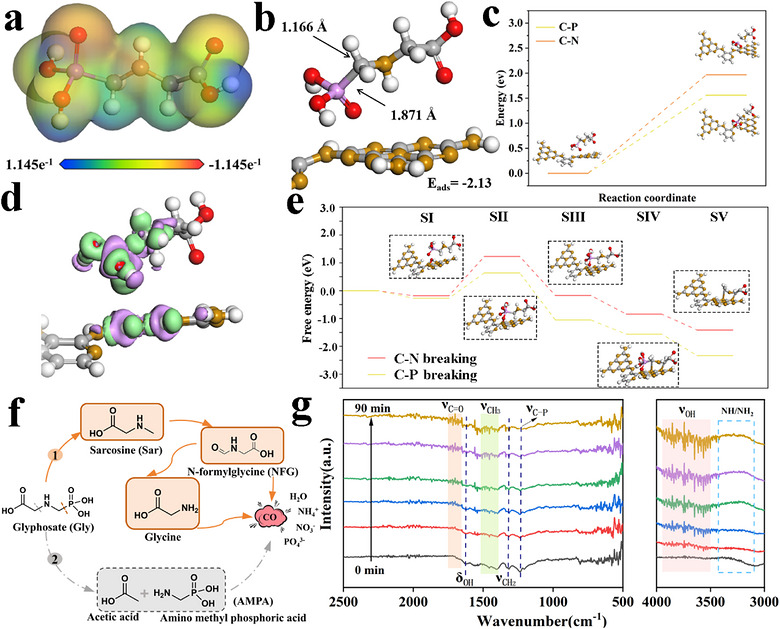
(a) Electrostatic potential map of the Gly molecule. (b) Schematic representation of Gly adsorption on PDCN. (c) The energy required to cleave the C─P and C─N bonds in the PDCN system. (d) Charge density difference of Gly adsorbed on PDCN (Purple and green represent the consumption and accumulation of electrons, respectively). (e) Free energy diagram for photocatalytic degradation of Gly with PDCN via the initial C─P and C─N bond cleavage pathways (f) Proposed reaction pathway for photocatalytic reforming of Gly to CO. (g) In situ FTIR spectra for the photocatalytic degradation of Gly on PDCN.

Fukui function analysis was employed to assess the reactive sites for radical attack toward Gly (Figure S17), and the results demonstrate that primary ROS (•OH) radicals tend to attack the N4 (ƒ^0^ = 0.1943) and O14 (ƒ^0^ = 0.1196) sites of the Gly molecule. Furthermore, the electrostatic potential map of Gly (Figure 7a) reveals a lower surface charge at the O14 site. The adsorption energies of Gly on the pyridine ring of PDCN and the heptazine ring of CN and PDCN catalysts were determined by DFT calculations to be −2.13, −0.49, and −1.65 eV, respectively (Figure S18). These findings demonstrate that the phosphate group of Gly exhibits a stronger adsorption affinity for the sp^2^ N sites on the heptazine ring of PDCN catalyst. Additionally, the adsorption energy of the −PO_3_H_2_ end of Gly on PDCN (−2.13 eV) is greater than that of the −COOH end (−1.09 eV) (Figure 7b; Figure S19). Compared to the original Gly molecule, the α‐C─H bond at the −PO_3_H_2_ terminus of adsorbed Gly elongates from 1.099 to 1.166 Å, with the C─P bond elongating from 1.810 to 1.871 Å (Figure S19a). Moreover, the HOMO‐LUMO of the PDCN‐Gly complex induced by light shows that electrons transfer from Gly to PDCN (Figure S20). These results indicate that −PO_3_H_2_ tends to adsorb onto PDCN through electrostatic interactions, which can lead to C─H bond activation. As the substrate ─COOH group adsorbs onto PDCN, a slight stretch of Gly's N─H bond is observed (Figure S19b), but the low adsorption energy renders N─H bond activation unfavorable. The energy barrier for the C─P bond cleavage is lower than that for C─N bond scission during photocatalytic degradation of Gly (Figure 7c), indicating that PDCN‐mediated photocatalytic degradation preferentially breaks the C─P bond of Gly. Further, the computed charge density difference of the Gly molecule adsorbed on PDCN (Figure 7d) shows a significant reduction in electron density at the ─COOH group, where electrons are accumulated through the phosphorus atom toward O14 of Gly and exhibit a propensity to transfer toward the heptazine unit of PDCN. The electrostatic interaction between the electron‐deficient heptazine ring and the electron‐rich −PO_3_H_2_ group of PDCN facilitates electron transfer, endowing great potential to activate the C─H bond of Gly and thereby achieve the selective cleavage of its C─P bond.

To investigate the mechanism and thermodynamics governing the selective cleavage of the C─P bond during Gly oxidation, the transition state energy barriers for both C─P and C─N bond cleavage pathways were calculated. As shown in Figure 7e, the nucleophilic attack of •OH on the P atom was identified as the rate‐determining step in the Gly oxidation process. This step facilitates accelerated electron transfer from the α‐carbon, leading to the formation of an unstable α‐carbon radical (SIII) [15]. Such a PCET process subsequently promotes the formation of the SIV transition state. Notably, the transition state free energy for the C─P bond cleavage pathway on PDCN is −2.33 eV, which is substantially lower than that of the C─N bond cleavage pathway (−1.41 eV). These results indicate that the initial C─P bond cleavage pathway exhibits higher selectivity than the C─N bond scission route during photocatalytic Gly degradation. To gain a deeper understanding of the catalytic mechanism of Gly, the degradation pathways of Gly were investigated (Figure 7f). Typically, the photodegradation of Gly primarily proceeds via two major routes: (i) initial cleavage of the C─P bond of Gly yields Sar, followed by decomposition to glycine, and (ii) the C─N bond cleavage of Gly is initiated to generate aminomethylphosphonic acid (AMPA) and acetic acid. Analysis of intermediate signal peaks during the photocatalytic degradation of Gly via the LC‐MS/HPLC and FTIR reveals the degradation pathway of Gly (Figure 7g; Figures S21 and S22, Table S3). LC‐M results indicate that Gly (m/z = 170) is the dominant species in the solution prior to irradiation. After 45 min of reaction, no Gly is detected, while intermediates such as Sar (m/z = 88) and N‐formylglycine (NFG, m/z = 102) are identified, likely because the C─P bond undergoes preferential cleavage to generate Sar, and some of the Sar is oxidized by reactive oxygen species to form NFG [61]. After 90 min, the content of glycine (m/z = 74) and Sar increases. Furthermore, quantitative HPLC analysis shows that the carbon mass balance of the products after 1.5 h of Gly photodegradation is 97.8%. The minor carbon loss is attributed to the adsorption of intermediates onto the catalyst surface. Meanwhile, the content of AMPA (0.006 mg, LOD = 0.206 µg L^−1^) accounted for less than 1% of the total products (Figures S21 and S22 and Tables S4 and S5), indicating that the PDCN photocatalytic system effectively avoids the formation of harmful intermediates during the photoreforming of Gly Combining thorough experimental and computational evidence, a feasible route can be outlined: first, the C─P bond in Gly cleaves to generate Sar, which then undergoes oxidation to afford NFG, followed by decarbonylation to yield glycine and release CO. Compared with the path of breaking the C‐N bond, the conversion of carbon resources into the usable gas CO avoids the generation of toxic AMPA. Further investigation into CO generation pathways using carbon‐containing intermediates such as glycine, Sar, NFG, acetic acid, and AMPA as substrates reveals that the Sar exhibits the maximum CO yield of 639.29 µmol gcat^−1^ h^−1^, which is 10.3 and 1.5 times greater than that of the AMPA (61.82 µmol gcat^−1^ h^−1^) and acetic acid (431.77 µmolgcat^−1^ h^−1^), respectively (Figure S23). These findings point to the initial C─P bond cleavage as the principal route through which Gly is transformed into CO.

After light exposure, the stretching vibration peaks of CH_2_ (ν_CH2_, 1314 cm^−1^) and C─P (ν_C‒P_, 1233 cm^−1^) in the FTIR spectra are gradually weakened, proving that the C─P bond at the terminal of Gly's ─PO_3_H_2_ group undergoes preferential cleavage (Figure 7g). Meanwhile, the peak of the methyl bending vibration (ν_CH3_, 1391‒1520 cm^−1^) is broadened, revealing the formation of the Sar intermediate. Under light exposure, the peak intensity of ν_OH_ (3500–3941 cm^−1^) corresponding to free hydroxyl groups shows a gradual increase, whereas the δ_OH_ peak (1627 cm^−1^) associated with O─H bending vibration exhibits a progressive decrease. Simultaneously, the peak intensity of aldehyde C═O stretching vibration (ν_C = O_, 1653–1753 cm^−1^) shows a steady increase, corresponding to the oxidation of Sar to generate the NFG intermediate. A gradual rise is observed in the NH_2_ stretching vibration peak (3094–3427 cm^−1^), which is associated with the formation of the glycine intermediate, as corroborated by LC‐MS/HPLC analyses (Figures S21 and S22). Based on the above characterization, it is illustrated that the active sp^2^ N sites of PDCN heptazine rings enhance the effective adsorption of Gly through electrostatic interaction, providing the driving force for the selective C─P bond cleavage of Gly by the •OH radicals generated from the electron‐rich pyridine moiety, thereby achieving the in situ upgrading of Gly to CO.

**FIGURE 8:**
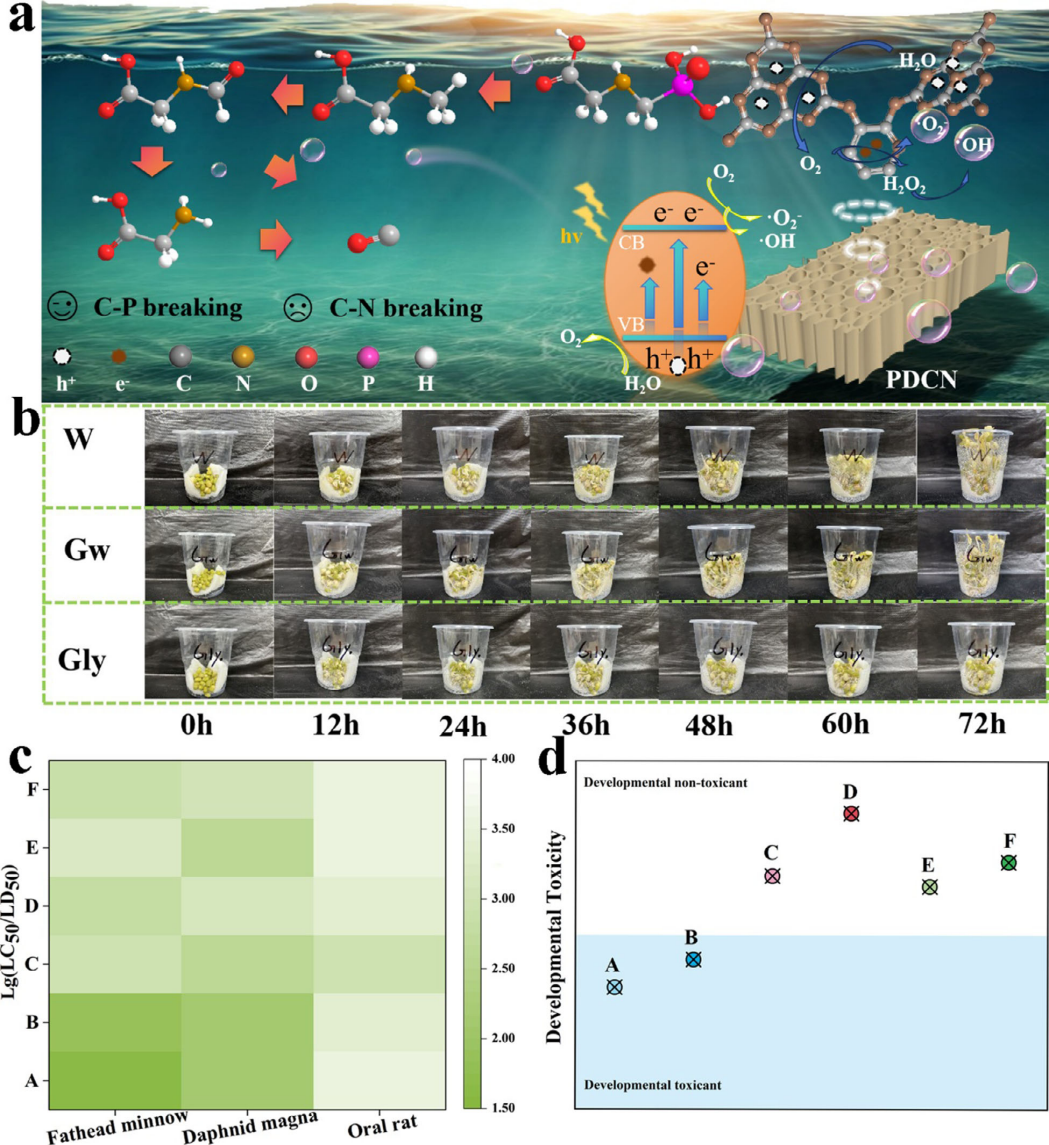
(a) Diagrammatic representation of the photocatalytic degradation pathway of Gly on PDCN. (b) Growth status of mung beans under different conditions after 72 h: W (deionized water); Gw (Gly degradation solution); original Gly solution (7.1 mmol L^−^^1^). (c) Acute toxicity (Lg) of AMPA, Gly, and their degradation products against Fathead minnow, Daphnia magna, and Oral rat as assessed by the T.E.S.T program, and (d) their developmental toxicity (A: Gly; B: AMPA; C: Sar; D: NFG; E: Glycine; F: Acetic acid).

Overall, a feasible mechanism for the photocatalytic CO evolution from Gly degradation with PDCN is proposed (Figure 8a). The asymmetric polar structure of PDCN mediates n‐*π* ^*^ electronic transitions through localized polarization fields (Figure 3a), enhancing photoresponsivity and synergizing with dipole fields to facilitate the dissociation and transfer of e^−^‒h^+^ pairs. The electron‐rich pyridine region (pyridine N site) of PDCN facilitates oxygen adsorption and generates substantial •OH. Furthermore, the phosphate group of Gly is preferentially adsorbed at the sp^2^ N sites on the edge of the PDCN heptazine ring via electrostatic interaction, where the α‐C─H bond of Gly is activated by a PCET process. The synergistic effect between ROS •OH and PCET process promotes selective C─P bond cleavage of Gly, thereby bypassing the AMPA intermediate. Ultimately, this process gives rise to a Sar intermediate, which undergoes further oxidation to produce gaseous fuel CO.

### 2.5 Environmental Assessment

To evaluate the ecotoxicity of Gly and its transformation products, the growth of mung bean sprouts was used as a toxicity indicator to observe the effects of Gly wastewater before and after degradation on the growth of mung bean sprouts. Solutions containing Gly at concentrations identical to those in the degradation experiments were used to cultivate mung beans [62]. The temporal growth process of mung bean sprouts is visually recorded in Figure 8b. Comparative analysis of growth under different culture media (Figure S24) reveals that, under ambient influences of temperature, humidity, and sprout orientation, the germination rate reaches approximately 76% in W and 65% in Gw, both demonstrating high germination efficiency. In contrast, virtually no germination occurs in the Gly solution. The number of sprouts attaining lengths of 5‒9 cm in Gw is half that in W, yet the growth trend closely mirrors that of W. When compared to sprouts in degraded Gly solution (Gw), those in Gly solution show significantly inhibited growth, suggesting that photocatalytic treatment greatly diminishes the toxicity of Gly.

To further evaluate the ecological toxicity of Gly and its transformation products, toxicity assessment software tools (T.E.S.T.) and quantitative structure‐activity relationship (QSARs) methodologies were adopted [61, 63]. In the photocatalytic PDCN system, the median lethal concentration (LC_50_) values of C (Sar), D (NFG), and E (glycine) for both Fathead minnow and Daphnia magna were lower than that of Gly (Figure 8c), indicating their non‐toxic nature. Furthermore, developmental toxicity assays (Figure 8d) demonstrate that most intermediates in the PDCN system exhibit no developmental toxicity. Toxicity evaluations indicate that the photocatalytic methodology developed via PDCN significantly reduces the toxicity of Gly, while simultaneously enabling the conversion of carbon waste into gaseous fuel CO. This approach aligns with the principles of environmentally sustainable water treatment.

## 3 Conclusion

In summary, a rational molecular design strategy is demonstrated for constructing high‐performance carbon nitride‐based photocatalysts through the self‐assembly of polar pyridine units on carbon nitride supports. The resulting electronic asymmetry and surface polarization, as confirmed by KPFM and dipole moment calculations, establish internal driving forces that significantly enhance charge carrier separation and migration. The spatially resolved electronic structure of the catalyst, characterized by electron‐deficient heptazine sp^2^ N sites and electron‐rich pyridine domains, enables dual‐functional photocatalytic pathways: electrostatic adsorption and activation of glyphosate via proton‐coupled electron transfer, alongside localized generation of reactive oxygen species (•OH) for selective C─P bond cleavage. The complete degradation of glyphosate within 1.5 h, coupled with a CO production rate of 1166.2 µmol g^−1^ h^−1^, which is superior to most reported relevant photocatalytic systems, underscores the catalyst efficiency and potential for converting organic pollutants into value‐added gaseous fuel. Importantly, comprehensive toxicity assessments confirm the environmental safety of the degradation products, without observing ecotoxicological effects on aquatic organisms or terrestrial plants. This work not only offers mechanistic insights into the molecular‐level regulation of non‐metallic photocatalysts, but also establishes a sustainable platform for wastewater remediation integrated with carbon‐based energy recovery, advancing the development of green chemistry and circular economy paradigms.

## 4 Experimental Section

### 4.1 Preparation of CN and PDCN

The synthesis of PDCN was performed using a conventional thermal polycondensation method [25]. In a mortar, 2 g of melamine and 0.16 g of 2,3‐diaminopyridine (DPY) were added and thoroughly ground to ensure uniform mixing. The well‐mixed precursors were then transferred into an Al_2_O_3_ crucible and placed in an air muffle furnace. The temperature was ramped up at 10 °C per minute to 550 °C, which was maintained for 2 h. After cooling down to room temperature, the resulting solid was taken from the muffle furnace and pulverized into a fine powder in a mortar, which is designated as PDCN. For the preparation of CN, melamine was used as the sole precursor, and the subsequent steps are identical to those described above (Figure S25).

### 4.2 Glycine Degradation and Analytical Method

Typically, 30 mg of photocatalyst was added to a quartz reaction tube containing 5 mL of Gly solution (7.1 mmol/L), and the mixture was stirred continuously at room temperature for a dark reaction of 30 min. Then, the solution was irradiated with a 300 W xenon lamp (λ ≥420 nm) for 90 min. The intermediates and products generated during the photocatalytic Gly degradation process were identified by liquid chromatography‐mass spectrometry (LC‐MS) (Table S3). The analysis was performed on an AB Sciex 4500 Trap LC‐MS system equipped with an ACQUITY UPLC BEH Shield RP18 column (100 mm × 2.1 mm × 1.7 µm) and an electrospray ionization source (AB Sciex Technologies, Framingham, USA). The concentrations of CO and O_2_ formed during Gly degradation were quantified by gas chromatography (GC) using a Fuli GC9790 instrument, which features a thermal conductivity detector (TCD) and a flame ionization detector (FID).

### 4.3 Toxicity Test

A controlled experimental framework was adopted, with mung beans serving as biological indicators, to evaluate the variations in the toxicity of organic pollutants before and after the photodegradation [62]. The specific steps are as follows: First, the mung beans were soaked in deionized water for 8 h. Then, the soaked mung beans were divided into three equal groups, and each group containing 38 mung beans was placed into a separate cup for the experiment. For the purpose of minimizing sunlight contact, the cups were covered with black cloth first and then stored in an area with sufficient ventilation. Every 12 h, each group was irrigated with deionized water (W), 7 mmol/L Gly solution (Gly), and Gly degradation product solution (Gw), respectively. The growth status of mung bean sprouts was regularly recorded to evaluate the impact of photodegradation on the reduction of pollutant toxicity. Comprehensive details regarding the detection conditions, calculation methods, and characterization techniques are provided in the Supporting Information section.

## Conflicts of Interest

The authors declare no conflicts of interest.

## Supporting information




**Supporting File**: advs75130‐sup‐0001‐SuppMat.docx.

## Data Availability

The data that support the findings of this study are available from the corresponding author upon reasonable request.
