# Supplementary material for: Polar Lattice‐Distorted Motifs Enable Synergy of Local Polarization/Dipole Fields for Concurrent Glyphosate Wastewater Remediation and CO Evolution
Source: Adv Sci (Weinh). 2026 Apr 2;13(36):e21941. doi: 10.1002/advs.202521941 (PMC13317577; doi:10.1002/advs.202521941)
Supplement: Supplementary file 1 — Supporting File: advs75130‐sup‐0001‐SuppMat.docx. [file ADVS-13-e21941-s001.docx]

### Supporting Information

### Polar Lattice-Distorted Motifs Enable Synergy of Local Polarization/Dipole Fields for Concurrent Glyphosate Wastewater Remediation and CO Evolution

*Daoping Chen, Huan Liu, Tengyu Liu, Jie Li, Qizhi Luo, Yan Zhang*, Shengkun Li*, and Hu Li**

D. Chen, H. Liu, T. Liu, J. Li, Q. Luo, Prof. Dr. S. Li, Prof. Dr. H. Li

State Key Laboratory of Green Pesticides, State-Local Joint Laboratory for Comprehensive Utilization of Biomass, Center for R&D of Fine Chemicals, Guizhou University, Guiyang, Guizhou 550025, China.

E-mail: hli13@gzu.edu.cn; skl505@outlook.com

Dr. Y. Zhang

Anhui Provincial Key Laboratory of Advanced Catalysis and Energy Materials, School of Chemistry and Chemical Engineering, Anqing Normal University, Anqing 246011, China; State Key Laboratory of Marine Resource Utilization in South China Sea, Department of Materials Science and Engineering, Hainan University, Haikou 570228, China. E-mail: zyan@hainanu.edu.cn

**Chemicals and materials**

Glyphosate (Gly, ≥98.0%), melamine (C_3_H_6_N_6_, ≥99.9%), 2,3-diaminopyridine (DPY, 96%), ethylenediaminetetraacetic acid (EDTA, 99.5%), potassium dichromate (K_2_Cr_2_O_4_), and catalase (CAT) were bought from Shanghai Aladdin Biochemistry Technology Co., Ltd. Isopropanol (IPA) and ethanol (99.7%) were purchased from TCI (Shanghai) Development Co., Ltd. All the chemicals used were not subjected to further purification or treatment. The aqueous solutions in this study were prepared using deionized water.

**Catalyst characterization**

The phase and morphology of all catalysts were investigated by scanning electron microscope (SEM, ZEISS Sigma 300), transmission electron microscopy (TEM, JEM-F200), and X-ray diffractometer (XRD, Bruker D8 Advance). UV-vis spectra were collected using a U-4100 spectrophotometer from Japan Hitachi with a diffuse reflectance Accessory. Brunauer-Emmett-Teller surface area (BET) was performed in the nitrogen adsorption apparatus (Micromeritics ASAP 2460). The chemical environment of carbon atoms was analyzed by solid-state ^13^C nuclear magnetic resonance (^13^C NMR, BRUKER AVANCE 400). The fluorescence emission intensity and time-resolved fluorescence spectrum of all samples were recorded using Edinburgh FLS1000. X-ray photoelectron spectroscopy (XPS) was utilized to investigate the valence states of those elements that were measured on a Thermo Scientific K-Alpha measurement with an Al Kα source. The O_2_ adsorption on the samples was performed by temperature programmed desorption (TPD, Micromeritics AutoChem II 2920). Fourier transform infrared (FT-IR) spectra measurements were conducted using a Thermo Fisher Scientific Nicolet iS20 FT-IR spectrometer in the wavenumber range of 400 to 4000 cm^‒1^. Photoelectrochemical properties were studied through photocurrent response and electrochemical impedance spectroscopy (EIS) measurements (Shanghai Chenhua-CHI660e). The electron spin resonance (ESR) signals of the samples were recorded on the Bruker EMXplus-6/1 spectrometer, when 5,5-dimethyl-1-pyrroline N-oxide (DMPO) or 2,2,6,6-tetramethylpiperidine (TEMP) was used as a trapping agent to capture reactive oxygen species under dark and illumination. Kelvin probe force microscopy (KPFM) measurements were carried out on a Bruker Dimension Icon AFM using a conductive probe (SCM-PIT-V2). Finally, the photo-assisted KPFM measurement was also conducted in the AFM test system equipped with a Xenon lamp.

**Photocatalytic tests**

The study examined the photocatalytic decomposition of Gly in aqueous medium utilizing a 300 W xenon lamp as the illumination source. In detail, the photocatalyst (0.030 g), along with Gly (5 mL, 7.1 mmol/L), was introduced into a quartz vessel (20 mL) and agitated at 25 °C for 30 min in the absence of light to attain complete adsorption equilibrium. Subsequently, the mixture was subjected to constant stirring for 1.5 h under simulated solar irradiation. For analytical purposes, the resultant solution was processed through filtration or centrifugation employing a 0.22 μm syringe membrane filter. Isopropanol (IPA, 10 mM), benzoquinone (BQ, 10 mM), K_2_Cr_2_O_7_ (10 mM), catalase (CAT), and ethylenediaminetetraacetic acid (EDTA, 10 mM) were introduced as scavengers for radicals ·OH, ·O_2_^−^, e^−^, H_2_O_2_, and h^+^, respectively. Furthermore, the stability of the photothermal catalyst was assessed through five consecutive cycling tests. Following each cycle, the catalyst underwent centrifugation, followed by rinsing with ethanol (15 mL × 3) and drying for the next test.

To assess the CO content during Gly degradation, the photocatalyst (0.030 g) was incorporated into the Gly solution (5 mL, 7.1 mmol/L), sealed, and agitated for 30 min devoid of light exposure. Subsequently, the mixture was continuously agitated for 100 min under simulated solar irradiation. The procedure for determining the O_2_ content during the Gly degradation is identical to that employed for CO assessment.

**Catalyst recyclability**

Upon completion of each experimental trial, the catalyst was recovered by centrifugation, subjected to alternate washing with ethanol and deionized water thrice, and subsequently dried in an oven at 60 ℃ for reuse. The Gly efficiency (ɧ) was determined utilizing the equation provided below.

ɧ = C_t_ / C_0_×100% (1)

Where C_0_ represents the initial theoretical concentration of Gly (mmol L⁻¹), and Cₜ denotes the Gly concentration at a given time (mmol L⁻¹).

The photocatalytic reaction mechanism of Gly can be characterized by the Langmuir-Hinshelwood kinetic model, representing it as an apparent pseudo-first-order kinetic equation (2).

−lnC_t_ / C_0_＝ kt (2)

Where k is the apparent pseudo-first-order rate constant.

**Density functional theory calculations**

Density functional theory (DFT) calculations were performed using the CASTEP method. ^[S1]^ The initial crystal structures employed in this study were sourced from the Materials Project database. The first principles were employed to perform all density functional theory (DFT) calculations within the generalized gradient approximation (GGA) using the Perdew-Burke-Ernzerhof (PBE) formulation. ^[S2]^ A plane-wave energy cutoff value of 500 eV was consistently employed throughout all computational analyses. The consistency tolerances for the geometry optimization were set as 0.001eV for total energy and 0.3 eV/Å for force. To prevent interactions between periodic slabs, a vacuum spacing of 20 Å was implemented. The self-consistent field (SCF) cycles were limited to a maximum of 500.

Finally, the adsorption energy (E_ads_) was defined as follows:

E_ads_ = E_ad/sub_ ‒ E_ad_ ‒ E_sub_

Where E_ad/sub_, E_ad_, and E_sub_ indicate the total energy for the adsorbate/substrate system, the energy of the adsorbent, and the energy of the adsorbed molecules, respectively.

**Statistical Analysis**

All experiments were performed independently in triplicate (n=3), and the results were expressed as mean ± standard deviation (mean ± SD). The photocatalytic degradation process follows the pseudo-first-order kinetics model. Statistical analysis was conducted using Origin software, and one-way analysis of variance (one-way ANOVA) was employed to evaluate differences between groups. Data fitting and kinetic analysis were also implemented using Origin, and a coefficient of determination R^2^ > 0.95 was considered indicative of satisfactory fitting.


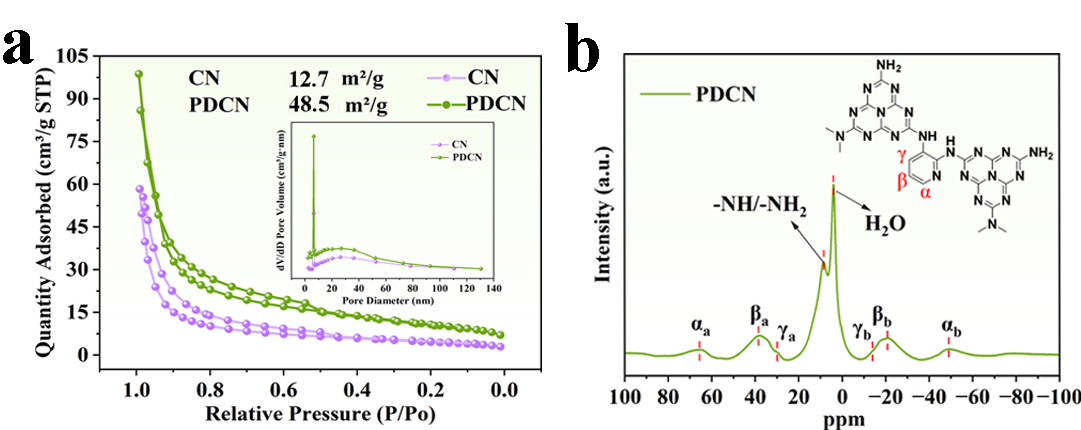


**Figure S1**. (a) N₂ adsorption-desorption isotherms of CN and PDCN (inset: pore size distribution). (b) The solid-state ^1^H NMR spectra of PDCN.


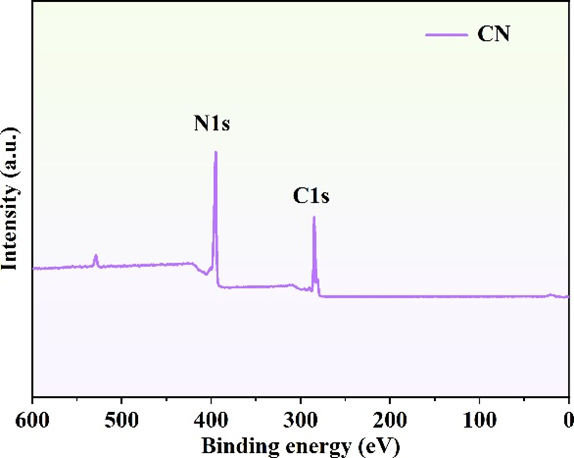


**Figure S2**. XPS survey spectrum of CN.


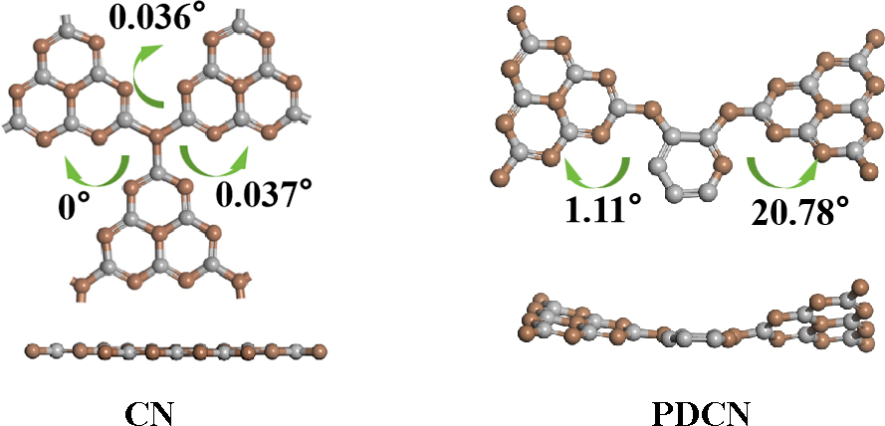


**Figure S3.** DFT geometric optimization and dihedral angles of CN and PDCN fragments.


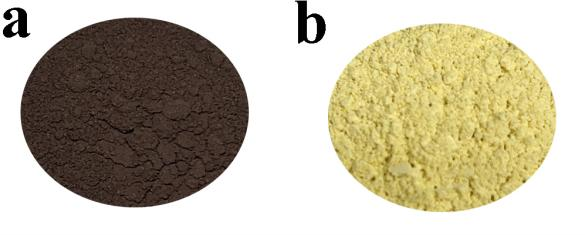


**Figure S4.** Colors of (a) PDCN and (b) CN catalyst powders.


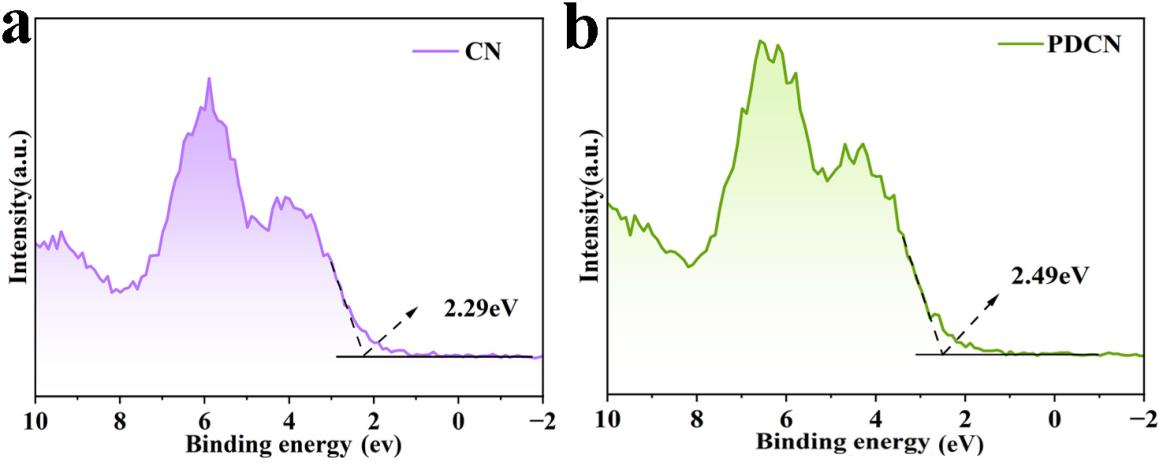


**Figure S5.** VB-XPS spectra of CN and PDCN.

E_VB_ = E_VBM_ + Φ ‒ 4.44eV (Equation S1)

E_g_ = E_CB_ + E_VB_ (Equation S2)

E_VB_, E_VBM_, Φ, E_CB_, and E_g_ represent the valence band, the maximum value of the potential valence band (from XPS), the work function of the instrument (4.2 eV), the conduction band potential, and the band gap, respectively.


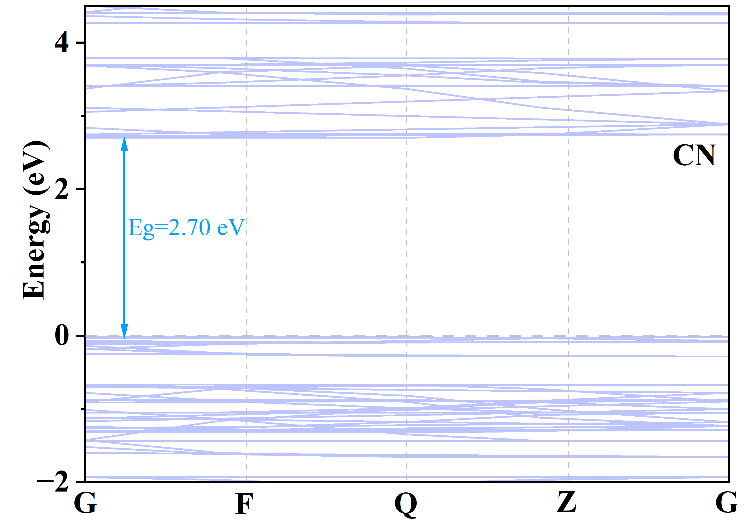


**Figure S6.** The DFT-calculated band diagrams of CN.


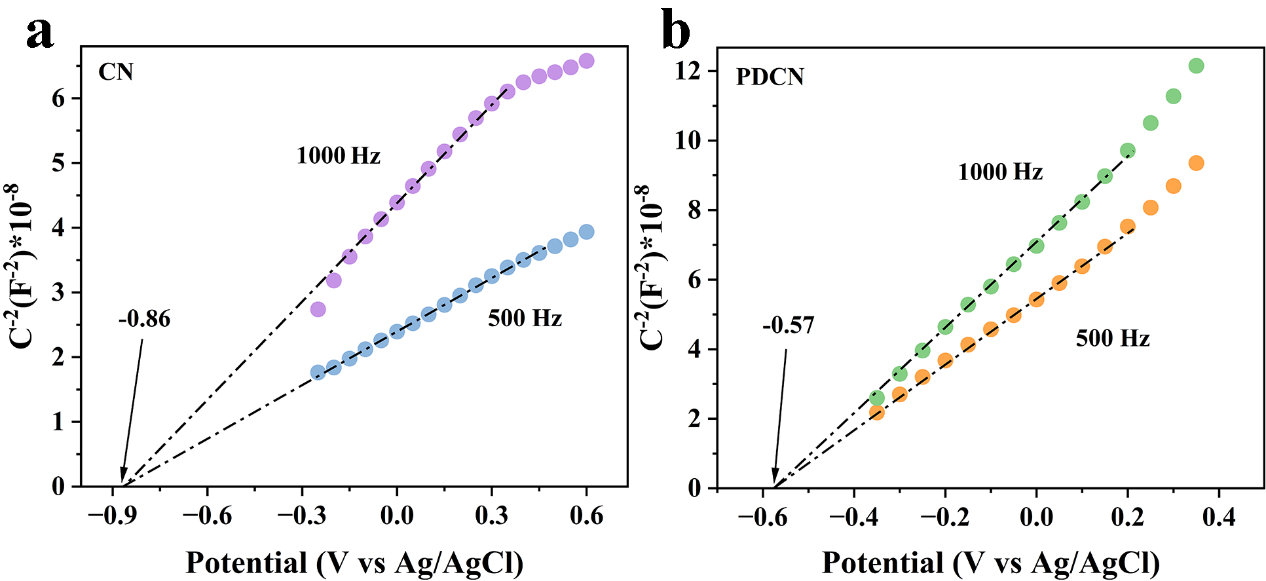


**Figure S7.** The M-S plots of CN and PDCN


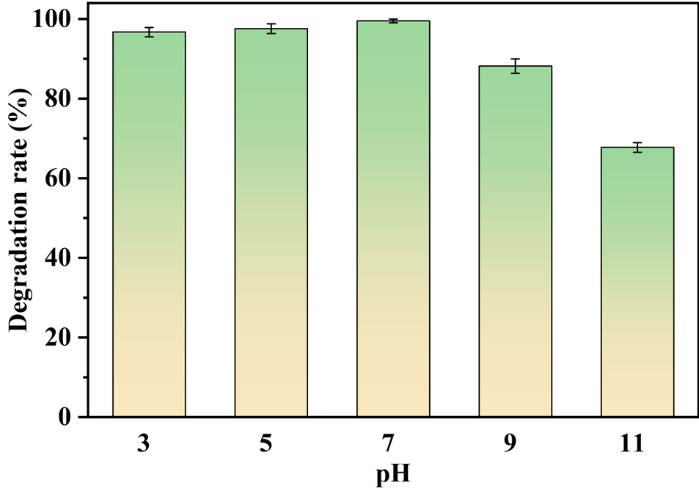


**Figure S8.** The impact of varying initial pH environments on Gly removal.


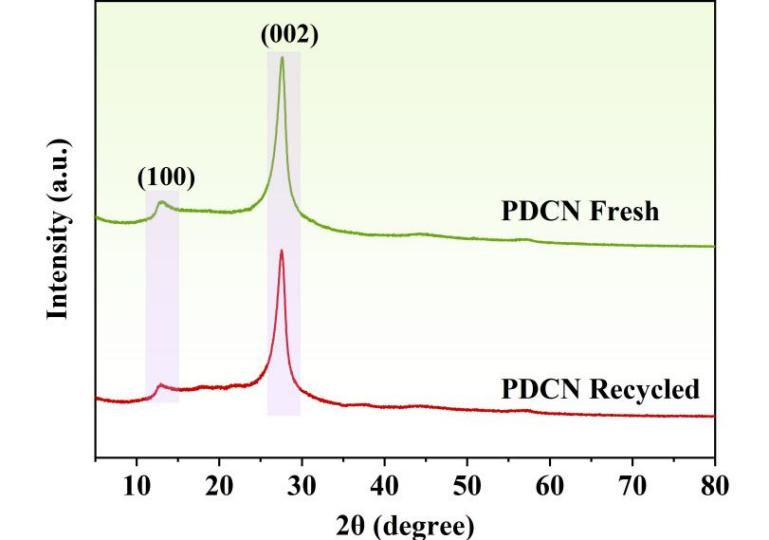


**Figure S9.** XRD patterns of fresh and recycled PDCN.


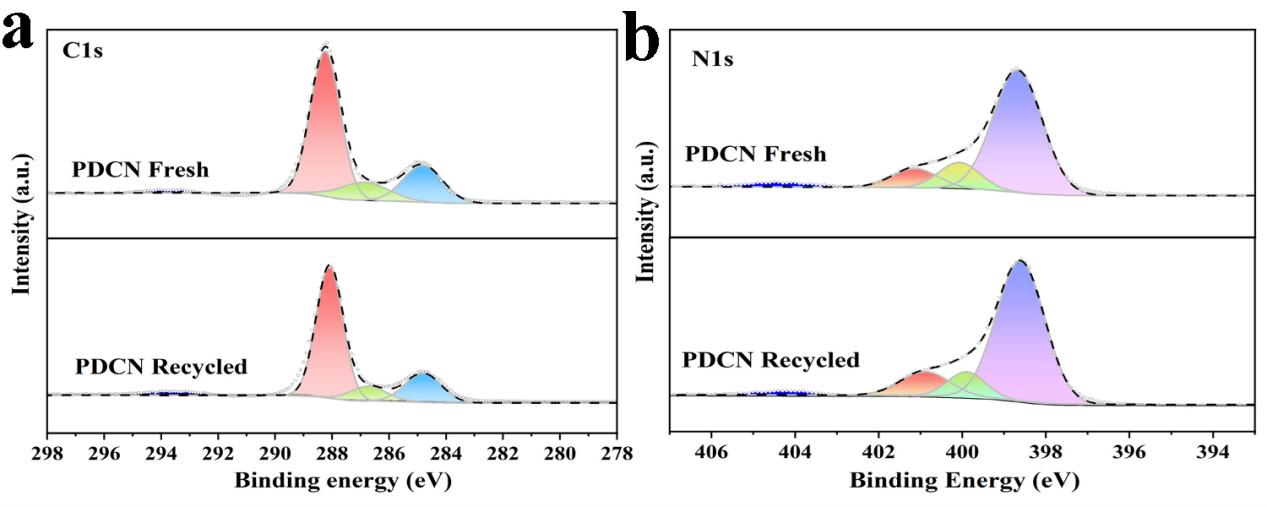


**Figure S10**. (a) C 1s and (b) N 1s XPS spectra of fresh and recycled PDCN.


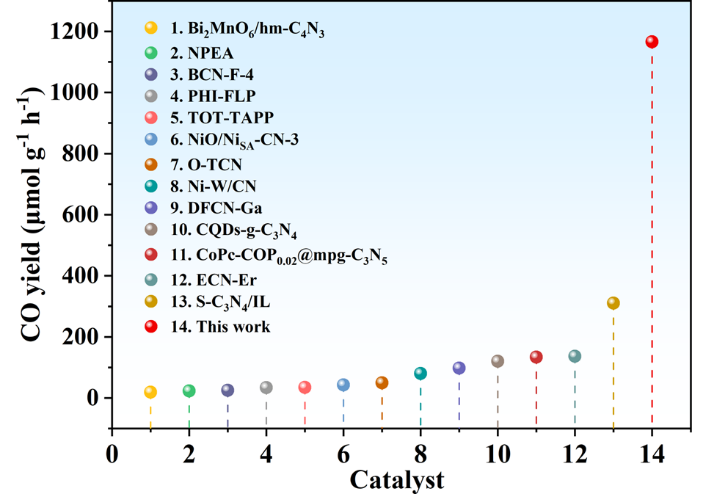


**Figure S11**. The photocatalytic CO_2_-to-CO reduction performance of different catalysts reported. ^[S3-S15]^


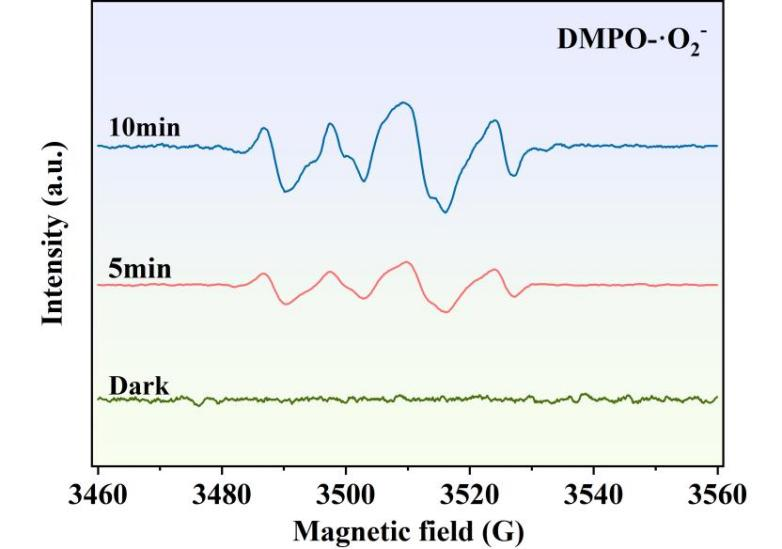


**Figure S12**. The DMPO-·O_2_^-^ ESR spectra of the PDCN catalyst.


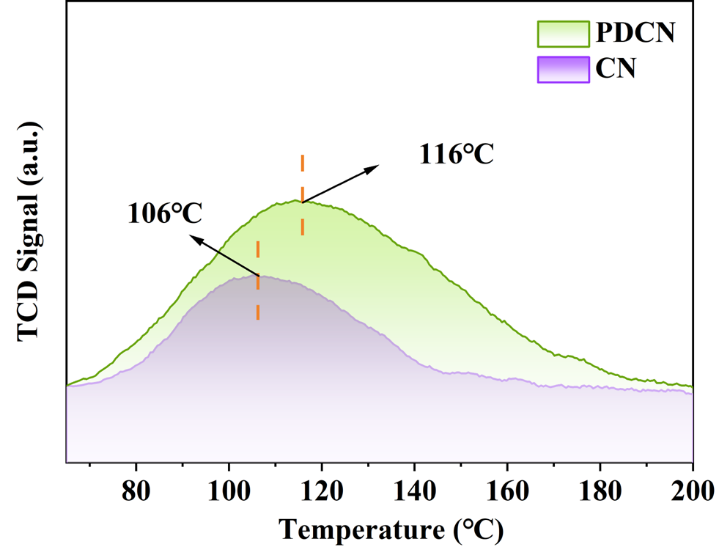


**Figure S13.** O_2_-TPD curves of PDCN and CN.


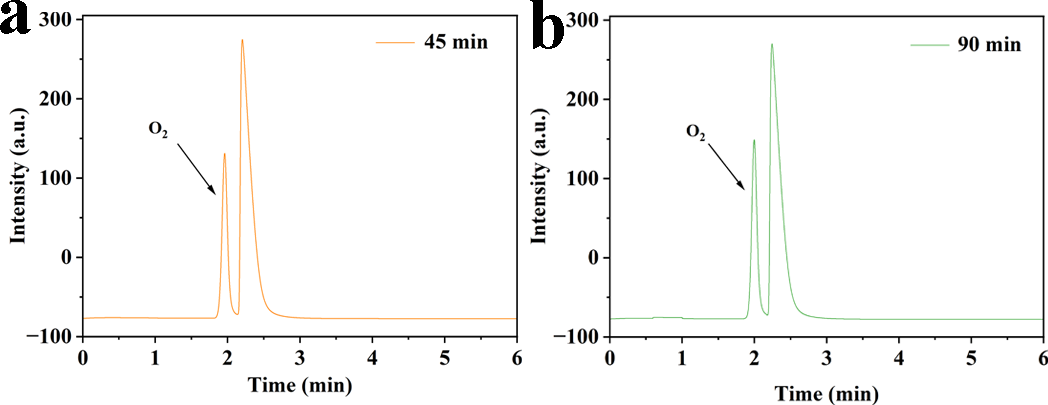


**Figure S14.** O_2_ detection with GC at (a) 45 min and (b) 90 min in PDCN-catalyzed reaction systems.


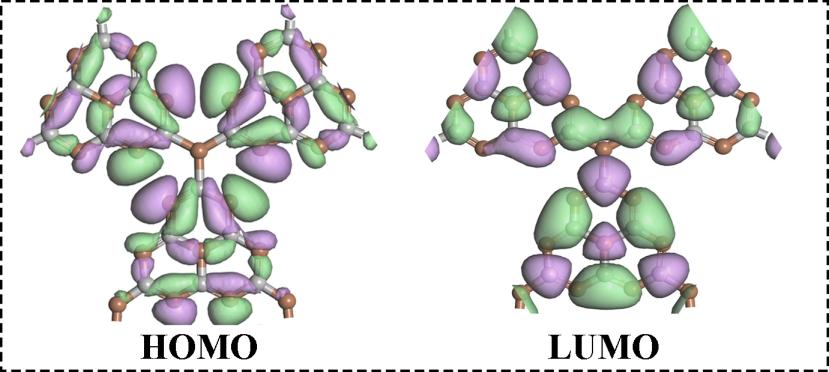


**Figure S15.** Distribution of HOMO and LUMO of CN.


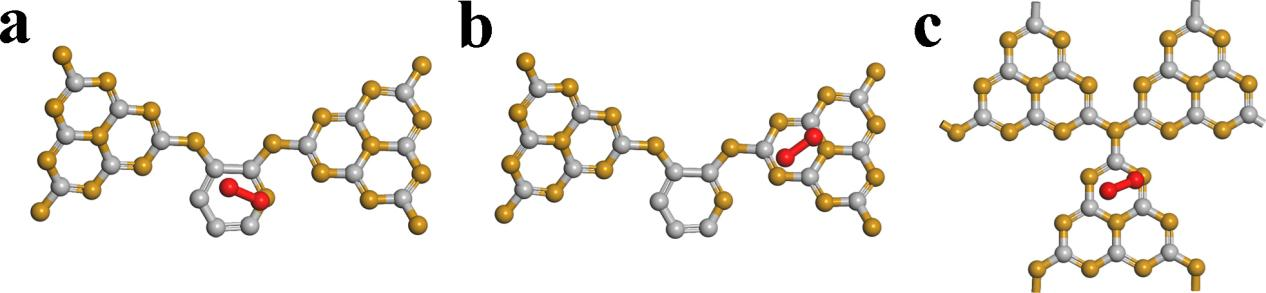


**Figure S16.** Modeling of O_2_ adsorption on (a) PDCN pyridine ring, (b) PDCN heptazine ring, and (c) CN.


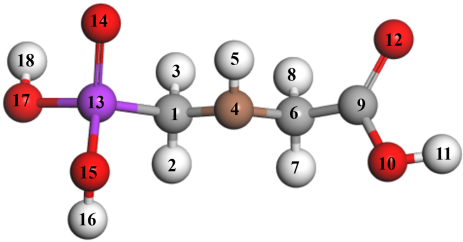

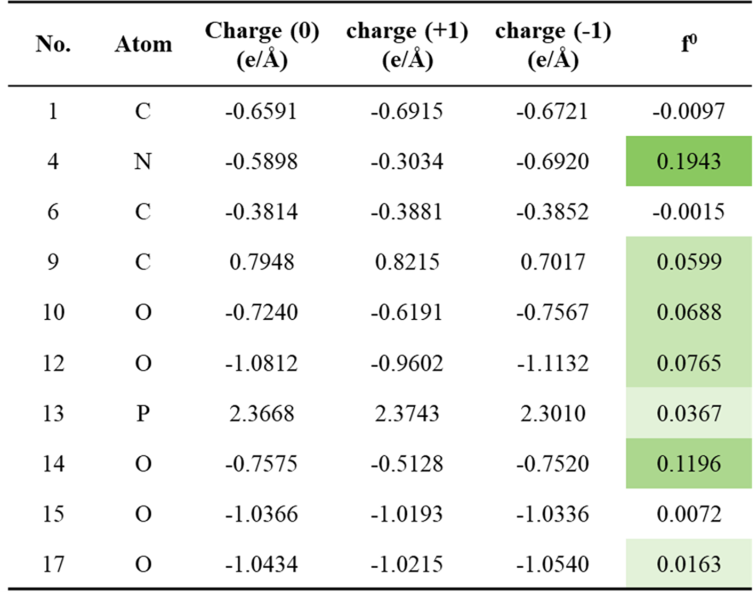


**Figure S17.** Calculated Fukui index of Gly.


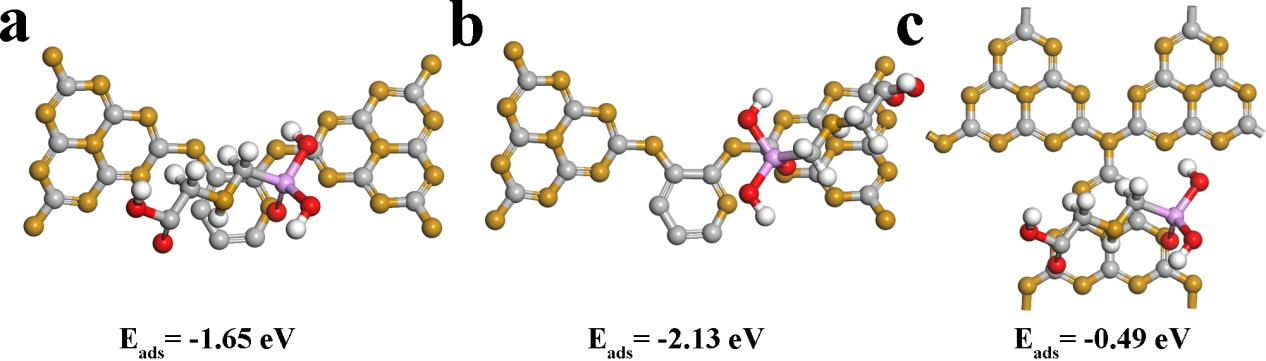


**Figure S18.** (a) Simulation of Gly adsorption on the pyridine ring of PDCN, (b) on the heptazine ring of PDCN, and (c) on CN.


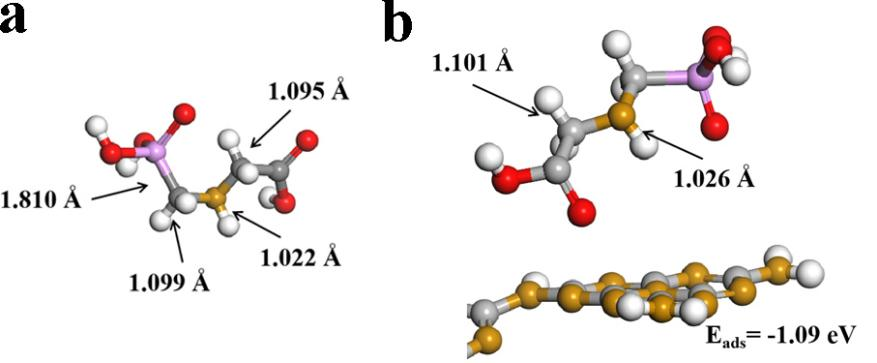


**Figure S19.** (a) Simulation of the optimized molecular model of Gly, and (b) the adsorption configuration of Gly at its ‒COOH terminus on the pyrazine ring of PDCN.


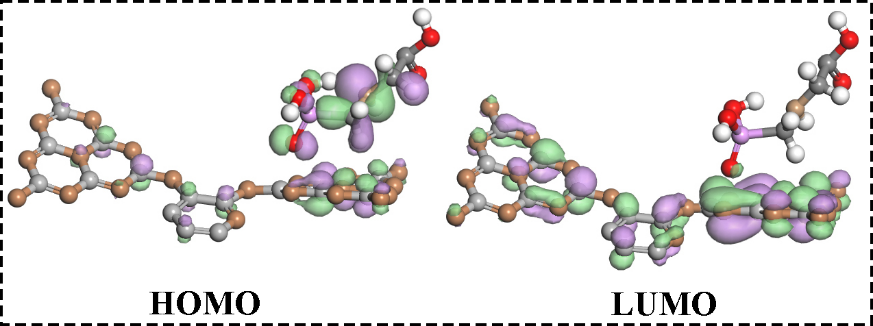


**Figure S20.** Electron distribution of the HOMOs and LUMOs in the photoinduced PDCN-Gly complex.


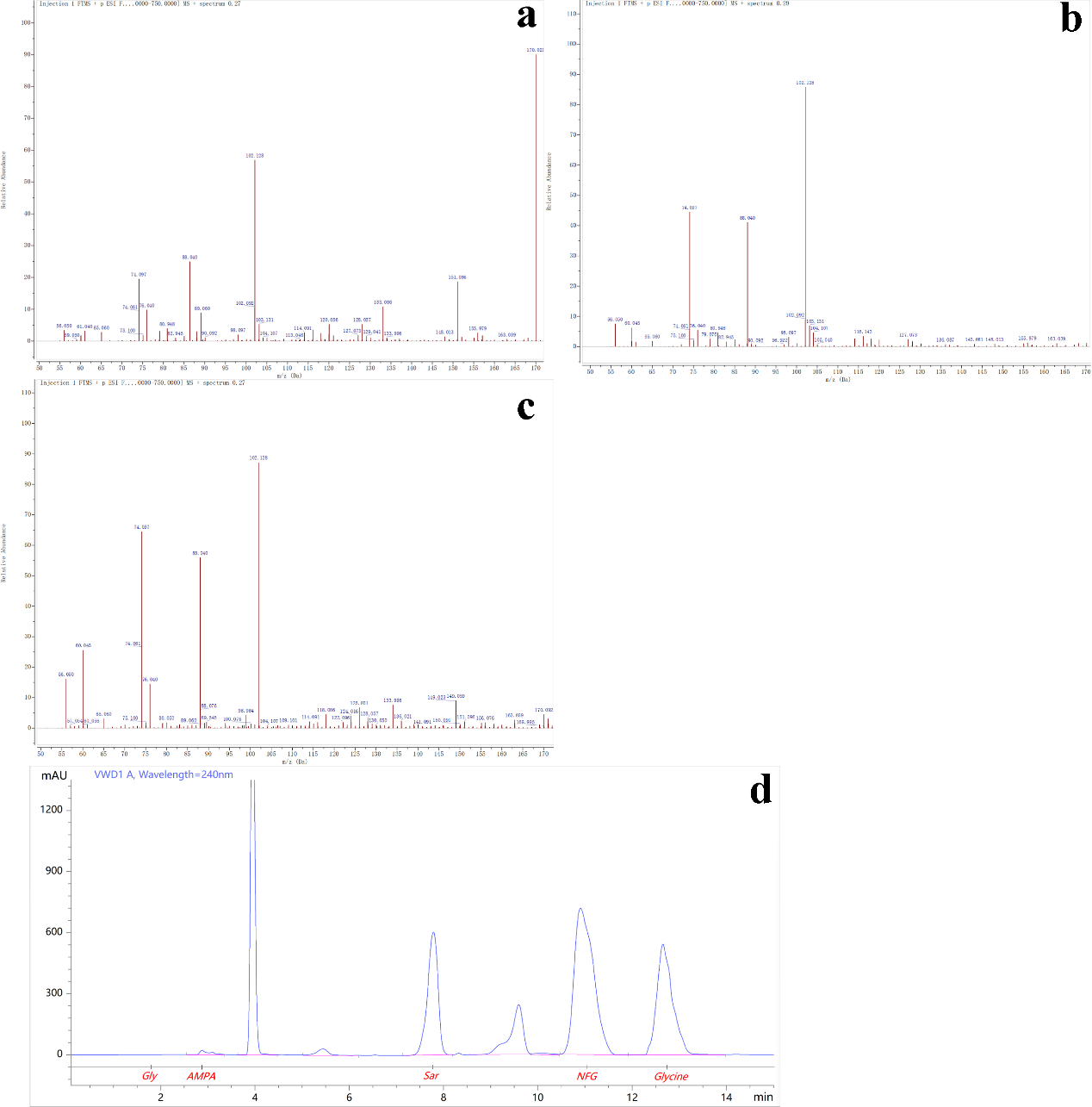


**Figure S21.** (a-c) LC-MS spectra of Gly degradation solution catalyzed by PDCN at 0 min, 45 min, and 90 min. (d) HPLC spectrum of Gly degradation solution catalyzed by PDCN at 90 min.


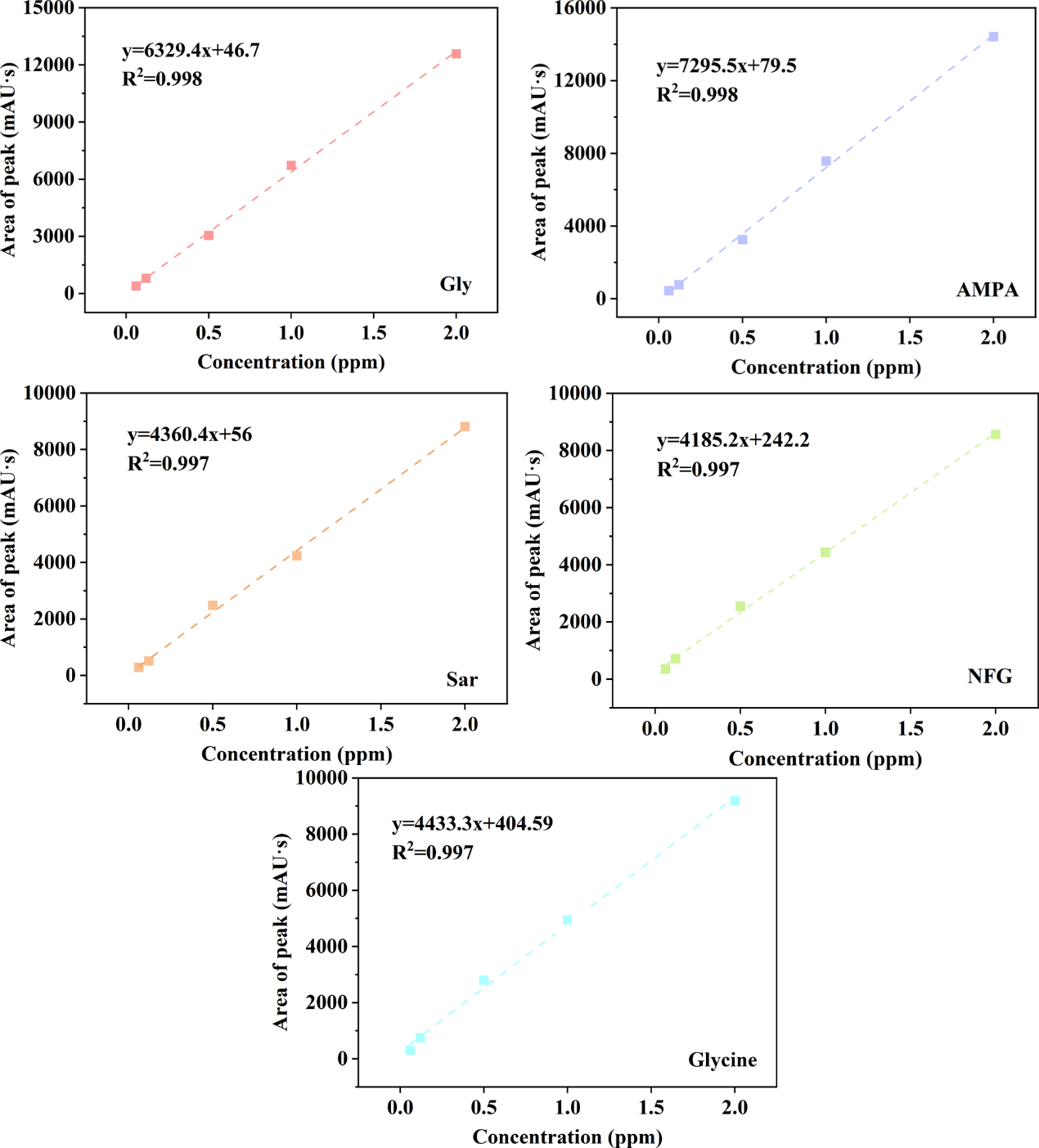


**Figure S22.** Standardization curves of Gly, AMPA, Sar, NFG, and Glycine.


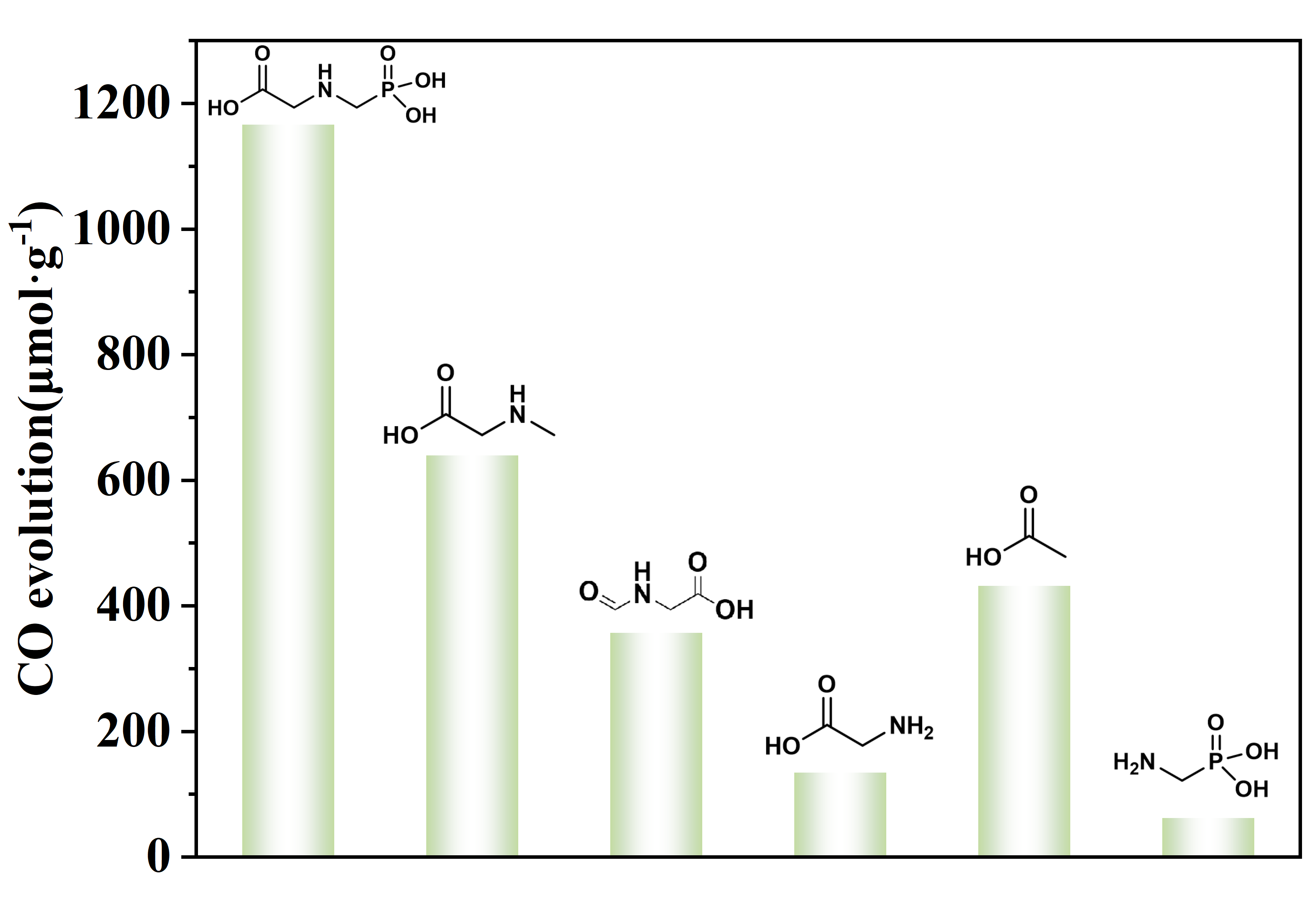


**Figure S23.** The CO release curves of Gly and its photodegradation intermediates. Reaction conditions: 30 mg PDCN catalyst, 0.1 mmol intermediate, 5 mL H_2_O, 300 W xenon lamp, 25 °C, 1 h.


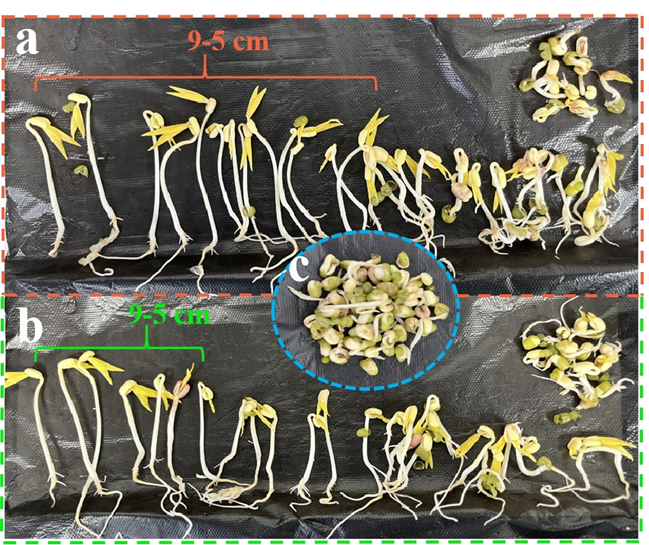


**Figure S24.** Growth of mung bean seeds after 72 h in deionized water (a) and solutions before (c) and after (b) photocatalytic degradation of Gly.


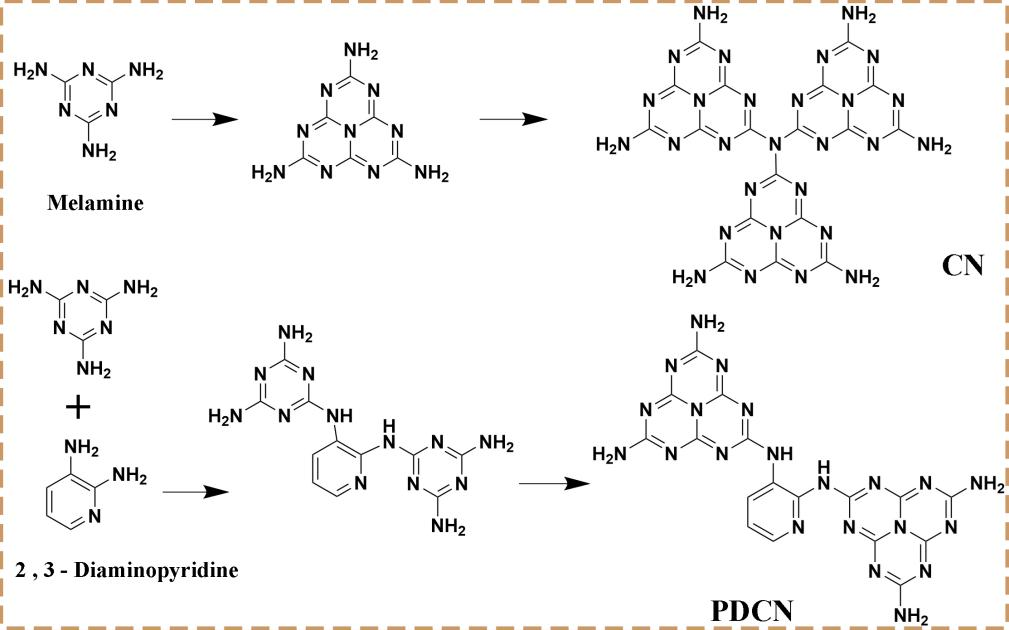


**Figure S25.** (a) Schematic diagram of the synthesis of CN and PDCN.

**Table S1**. Elemental composition (atom ratios) of all samples according to XPS analysis.

| Sample | C% | N% | C/N |
| --- | --- | --- | --- |
| CN | 46.14 | 53.86 | 0.86 |
| PDCN | 47.6 | 53.13 | 0.90 |

**Table S2**. TRPL fitting results of CN and PDCN.

|  | τ_1_/ns | τ_2_/ns | τ_avg_/ns |
| --- | --- | --- | --- |
| CN | 1.79 | 9.23 | 5.63 |
| PDCN | 0.84 | 3.94 | 2.41 |

**Table S3**. The intermediates formed in photocatalytic degradation of Gly with PDCN.

| Entry | Signal peak m/z | Species |
| --- | --- | --- |
| 1 | 170.021 | Glyphosate |
| 2 | 112.016 | Aminomethylphosphoric acid |
| 3 | 102.128 | N-formylglycine |
| 4 | 88.04 | Sarcosine |
| 5 | 74.097 | Glycine |
| 6 | 60.045 | Acetic acid |

**Table S4**. The calculated carbon mass balance during Gly degradation

| Item | | Content | Carbon mass (mg) | |
| --- | --- | --- | --- | --- |
| Feedstock carbon | | Glyphosate | 1.278 | |
| Product carbon | | Sarcosine | 0.181 | |
|  | | Glycine | 0.170 | |
|  | | N-Formylglycine | 0.248 | |
|  | | Aminomethylphosphoric acid | 0.006 | |
|  | | CO | 0.646 | |
| Total output carbon | |  | 1.251 | |
| Carbon balance |  | | | 97.8% |

Carbon balance (%) = (Total recovered carbon / Initial carbon) × 100%

**Table S5**. Limit of detection (LOD and linear equation of glyphosate and its metabolites.

| Item | Linear equation | R^2^ | LOD/(ppm) | LOD/(μg·L^‒1^) |
| --- | --- | --- | --- | --- |
| Glyphosate | y=6329.4x+46.7 | 0.998 | 2.37×10^‒4^ | 0.237 |
| Sarcosine | y=4360.4x+56 | 0.997 | 3.44×10^‒4^ | 0.344 |
| Glycine | y=4433.3x+404.59 | 0.997 | 3.38×10^‒4^ | 0.338 |
| N-Formylglycine | y=4185.2x+242.2 | 0.997 | 3.58×10^‒4^ | 0.358 |
| Aminomethylphosphoric acid | y=7295.5x+79.5 | 0.998 | 2.06×10^‒4^ | 0.206 |

The limit of detection (LOD) was calculated using the signal-to-noise ratio (S/N = 3) method. The formula applied was LOD = 3 × N/k, where N (N = 0.5 mAU) is the peak-to-peak baseline noise measured at the retention time of each analyte in a blank solvent chromatogram, and k is the slope of the corresponding calibration curve.

**References:**

1. C. Li, H. Wu, D. Zhu, T. Zhou, High-efficient charge separation driven directionally by pyridine rings grafted on carbon nitride edge for boosting photocatalytic hydrogen evolution [J], *Appl. Catal. B: Environ*. **2021**, 297, 120433.
2. Z. Hu, Y. Zhang, Y. Wang, J. Huang, S. Yang, H. Li, Regulation of oriented NO photooxidation to circumvent NO_2_ with high-coordinated M–N_5_ (M = Fe, Co, Mn, Ti) sites and far–carbon defects [J], *Appl. Catal. B: Environ*. **2024**, 350, 123948.
3. H. Zhou, M. Huang, Y. You, J. Dai, M. Bao, J. Duan, J. Yang, Z. Jiang, Near-Infrared Photothermal Effect Boosting Photocatalytic CO_2_ Reduction by Black Ga-Doped Carbon Nitride [J], *IEC Res* **2025**, 19, 9692-9700.
4. L. Chen, S. Du, Y. You, M. Yuan, G. Zhou, L. Zhao, Y. Li, L. Yao, Z. Jiang, Synergistic photothermal and photocatalytic contribution to efficient CO_2_ reduction by Er-doped echinus-like carbon nitride [J], *Chem. Eng. Sci.* **2025**, 316, 121990.
5. J. Rong, Q. Xu, J. Han, K. Ren, J. Zhang, X. Zhao, P. She, J. Qin, H. Rao, In situ construction of cobalt phthalocyanine covalent organic polymer on mesoporous graphitic carbon nitride for boosting photocatalytic CO_2_ reduction [J], *Chem. Eng. J*. **2025**, 507, 160814.
6. W. Hou, Y. Li, K. Wang, H. Guo, B. Zhang, L. Wang, Boosting photogenerated charge accumulation of oxidated carbon nitride nanotubes for efficient CO_2_ photoreduction [J], *Sci. China Chem* **2025**, 68, 5293–5301.
7. Q. Xu, S. Wang, Y. Wang, X. Wu, J. Dai, J. Liu, D. Fang, C. Zhang, S. Sun, T. Cheng, H. Yang, Enhanced photocatalytic CO_2_ reduction via S atom-promoted carbon nitride complexed with imidazolium-based ionic liquids: Achieving superior selectivity [J], *Sep. Purif. Technol.* **2025**, 367, 132888.
8. L. Li, X. Dai, C. Cheng, F. Chen, S. M. Wabaidur, Synergy of Ni single atoms and NiO nanoclusters in carbon nitride to create local charge polarization for enhanced CO_2_ photoreduction [J], W. Wang, Y. Hu, *Chem. Eng. J*. **2025**, 507, 160101.
9. G. Yan, B. Xu, R. Zhang, W. Teng, T. Zhou, H. Li, W. Hua, B. Lin, G. Yang, Noble-metal-free Ni-W dual-atom sites with bifunctional synergy for efficient photocatalytic CO_2_ conversion [J], *Appl. Catal. B: Environ.* **2025**, 378, 125535.
10. X. Zeng, H. Chen, W. Fang, Z. Huang, D. Wang, X. He, X. Du, W. Li, H. Zhang, L. Zhao, Local spatial polarization induced efficient electron transfer in fluorinated borocarbonitride for boosting CO_2_ photoreduction [J], *Chem. Eng. J.* **2024**, 488, 151042.
11. Z. Zhou, H. Zeng, C. Feng, L. Li, R. Tang, W. Li, Y. Huang, Y. Deng, Engineering an annular donor–acceptor reaction chamber with spontaneous feedstock collection for boosting CO_2_ photoreduction [J], *Energy Environ. Sci*. **2024**, 17, 5627-5638.
12. Y. Xu, W. Hou, K. Huang, H. Guo, Z. Wang, C. Lian, J. Zhang, D. Wu, Z. Lei, Z. Liu, L. Wang, Engineering Built-In Electric Field Microenvironment of CQDs/g-C3N4 Heterojunction for Efficient Photocatalytic CO_2_ Reduction [J], *Adv. Sci.* **2024**, 11, 2403607.
13. C. Zhu, K. Zhong, B. Zhu, S. Li, H. Li, J. Yang, H. Xu, Engineering the S-scheme heterojunction modulating the charge density of the central carbon atom of half-metallic carbon nitride for boosting CO_2_ photoreduction [J], *Appl. Catal. B: Environ.* **2025**, 371, 125200.
14. D. Zhou, Q. Chen, J. Zhang, T. Wang, Z.-Q. Liu, Ether-Embedded Covalent Organic Frameworks Enable Efficient Photocatalytic CO_2_ Reduction [J], *Angew. Chem. Int. Edit*. **2025**, 64, e202500329.
15. M. Zhou, H. Wang, R. Liu, Z. Liu, X. Xiao, W. Li, C. Gao, Z. Lu, Z. Jiang, W. Shi, Y. Xiong, Construction of frustrated lewis pairs in poly (heptazine imide) nanosheets via hydrogen bonds for boosting CO_2_ photoreduction [J], *Angew. Chem. Int. Ed*. **2024**, 63, e202407468.
